# Enhancing rural B&B management through machine learning and evolutionary game: A case study of rural revitalization in Yunnan, China

**DOI:** 10.1371/journal.pone.0294267

**Published:** 2024-03-28

**Authors:** Wiseong Jin, Kwisik Min, Xufang Hu, Shengchao Li, Xueqin Wang, Bodong Song, Chengmeng Li

**Affiliations:** 1 Development and Planning Department of North Sichuan Medical College, Nanchong, China; 2 Hanyang University, Seoul, South Korea; 3 Yichuan County Water Resources Bureau, Luoyang City, China; 4 Department of Chinese Language and Literature, Jeonbuk National University, Jeonju-si, Jeollabuk-do, South Korea; 5 The Chinese University of Hong Kong, Hong Kong, China; 6 Kunming University, Kunming, China; 7 Gachon University, Seongnam, South Korea; Fiji National University, FIJI

## Abstract

The rural B&B industry is a key component of rural tourism, local economic development, and the wider rural revitalization strategy. Despite the abundance of tourism resources in Yunnan, the B&B sector faces significant challenges. It is therefore imperative to accurately identify the most pressing issues within the current B&B industry and formulate appropriate solutions to advance Yunnan’s rural revitalization efforts. This study uses recent reviews of rural B&Bs on Ctrip.com and employs machine learning techniques, including Bert, CNN, LSTM, and GRU, to identify the key management challenges currently facing Yunnan’s rural B&B industry. An analysis is then conducted to identify the key stakeholders involved in the process of improving the management of Yunnan’s B&Bs. To assess the willingness of each stakeholder to support the improvement of the rural B&B industry, this paper establishes a three-party evolutionary game model and examines the dynamic evolutionary process of management improvement within Yunnan’s rural B&B industry. Two scenarios of evolutionarily stable strategies are analyzed, and parameters impacting stakeholders’ strategy choices are simulated and evaluated. The results show that: i) Improving the "human factor" is the top priority for the current management improvement because tourists are most concerned about the emotional experience. Operators need to focus on improving service attitude and emotional experience; ii) The main stakeholders in the current management optimization process of Yunnan B&Bs are the local government, B&B operators, and tourists. Under appropriate conditions, the evolutionarily stable strategy of (1, 1, 1) is reachable. iii) variables such as additional costs, tourists’ choice preferences, and government penalties significantly affect the strategy choices of stakeholders, especially B&B operators. This paper offers effective strategies for improving B&B management that can benefit the government, B&B operators, and tourists, and ultimately contribute to the promotion of quality rural revitalization. The paper not only identifies focal areas for improving B&B management in rural Yunnan, but also provides an in-depth understanding of stakeholder dynamics. As a result, it provides valuable insights to further the cause of quality rural revitalization.

## Introduction

Rural development has long been a priority for poverty alleviation and rural revitalization [1]. Recognizing the potential of rural tourism, the Chinese government has placed great emphasis on supporting and guiding farmers to participate in bed and breakfast (B&B) operations [2]. The natural scenery and cultural heritage of rural areas have attracted a growing number of tourists as China continues to modernize and grow economically, leading to a surge in the popularity of B&Bs as a new form of tourism [3, 4]. In Yunnan Province, which is rich in natural resources, tourism has played a crucial role in driving overall development, generating total tourism revenue of 523.299 billion yuan in the first half of 2019 alone, and ranking first in China for rural tourism resources and first in western China for distinctive neighborhoods and scenic agricultural, forestry, and animal husbandry areas [5]. B&Bs have become a significant contributor to rural tourism consumption, promoting local economies, cultural traditions, and protecting customs. Investing in rural tourism is key to attracting investment, reducing population outflow, and realizing rural revitalization strategies, especially in the post-epidemic era [6, 7].

Despite the popularity of B&Bs in Yunnan, the current level of operation and management falls far short of the quality standards required for successful rural revitalization. The majority of B&Bs are family-owned and operated by residents who lack business and tourism management skills [8], resulting in low-quality services that cannot meet the growing demand of tourists [9]. Because the B&B industry is still in its infancy, regulations, and legal systems are not sound, and B&B operators lack professional skills, resulting in poor management that negatively impacts the tourist experience. This, in turn, leads to a high level of negative public opinion about rural tourism in Yunnan, which discourages many tourists, thus affecting the development of the local tourism industry and hindering the revitalization of the countryside. While local government and B&B operators recognize the need for improvement, they are constrained by financial and human resource limitations that limit their ability to rapidly and comprehensively improve all facets of B&B facilities. As a result, they must prioritize the most pressing core issues. It is therefore of paramount importance to carefully identify the prevailing challenges faced by rural B&Bs in Yunnan and to implement management improvements aimed at addressing these identified problems. This is indeed the central theme and focus of this paper.

The rise of the Internet has led to an increasing reliance by tourists on travel websites for selecting and booking B&Bs [10]. In particular, B&B ratings and reviews, which are prominently featured on these websites, have a significant influence on tourists’ decision-making processes [11]. Consequently, this avenue provides an opportunity for timely identification and understanding of issues within B&B management. Specifically, the approach involves analyzing reviews on online platforms, extracting tourists’ concerns and identifying corresponding emotional inclinations. This method allows the identification of prevalent issues in rural B&Bs in Yunnan [12]. Remarkable advances in computer technology, particularly in the field of machine learning, have facilitated the collection and analysis of large amounts of textual data. For example, machine learning techniques have been used effectively to study consumer purchasing behavior and attitudes toward internet-famous cuisine [13]. In tourism, machine learning can also be used to investigate tourists’ perceptions and emotional inclinations toward destination brands [14]. At the same time, game theory has been widely used to analyze the evolution of strategic choices among stakeholders, providing valuable managerial insights to address perceived problems. For example, evolutionary game theory has been skilfully used to construct a three-party game model involving government agencies, innovation-supplying firms, and potential demand firms. This model has effectively examined the diffusion dynamics of green technological innovations within manufacturing firms [15]. Furthermore, game theory has contributed to the study of low-carbon technology sharing between advantaged and disadvantaged firms within collaborative innovation systems [16]. Scholars have also used evolutionary game theory to study the behavior of government agencies in the context of green development, and have subsequently formulated management recommendations based on the results of these evolutionary games [17].

Given this background, it is of paramount importance to explore the prevailing challenges in the management of B&Bs in Yunnan. In addition, there is a need to conduct an in-depth analysis of the evolutionary stabilization strategies adopted by various stakeholders throughout the optimization process of Yunnan B&B management, especially after a prolonged period of engagement in evolutionary games. Furthermore, it is crucial to identify the key determinants that influence these evolutionary stabilization strategies. This research has significant implications for the development of the B&B sector in Yunnan. Furthermore, the approach of identifying and solving current problems can be extrapolated to address problems in other regions. Therefore, this paper aims to answer the following questions: i) What are the main challenges currently facing B&Bs in Yunnan, and what factors influence tourists’ decisions to select or reject a particular B&B? ii) Who are the stakeholders involved in the process of optimizing the management of B&Bs in Yunnan, and how can their costs and benefits be quantified and structured into a benefit matrix? iii) How can the asymptotic stability of the strategies of these stakeholders be analyzed, and what conditions must be met to achieve Evolutionary Stable Strategies (ESS)? iv) What is the impact of critical variables on evolutionary results and trajectories?

The main objective of this study is to investigate the key challenges currently facing B&Bs in Yunnan and to provide management recommendations based on an evolutionary game approach. To identify these challenges, we have developed an analytical framework based on the latest data and applied a variety of machine learning algorithms to identify the most pressing issues in Yunnan B&B management. We then used evolutionary game theory to analyze these issues and formulate management recommendations based on the results derived from the machine learning framework. To elaborate our methodology, we first conducted web scraping of recent tourist reviews of Yunnan accommodation from Ctrip.com, China’s largest online travel platform. This corpus of reviews was meticulously analyzed using machine learning techniques to extract themes and perform sentiment analysis, both of which are proven methods for addressing real-world problems [18]. Next, the findings from our machine learning framework were scrutinized to identify the stakeholders involved in solving the most critical current challenges in Yunnan accommodation. Based on the actual circumstances, we made reasonable assumptions and constructed a benefit matrix. We then engaged in an extensive discourse on asymptotic stability and evolutionary stabilization strategies under different conditions. Finally, this paper culminates in numerical simulations of key parameters and provides more robust management recommendations for relevant stakeholders with the overall goal of improving the management of accommodation in Yunnan.

This paper contributes to the existing literature in several significant ways. Firstly, in the post-epidemic era, as the tourism industry experiences a rapid resurgence, the analysis of critical issues facing rural B&Bs and the formulation of management recommendations hold paramount importance for rural revitalization efforts. Secondly, we employ machine learning algorithms to analyze review texts thematically and sentimentally, allowing us to discern the most pressing challenges in the current management of Yunnan lodgings. Notably, our framework exhibits a higher accuracy rate compared to traditional machine learning algorithms. Thirdly, our study extends the application of evolutionary game models, marking the inaugural utilization of evolutionary game theory in the examination of rural B&B development in the post-epidemic era. The insights garnered from this research offer valuable theoretical guidance for the advancement of rural lodging and rural revitalization efforts. Fourthly, recognizing that the rural lodging industry holds global significance, we acknowledge the regional variations in its current state. The research framework, centered on issue identification and management recommendation development through the analysis of scraped reviews, can be readily applied to B&B management in diverse regions, thereby offering potential benefits for the development of other areas as well.

The remaining sections of this paper include a review of related research, an explanation of the machine learning techniques used, a framework for analyzing B&B management problems and the results of the analysis, an evolutionary game model and numerical simulations for improving B&B management, and management recommendations for promoting high-quality B&B development in Yunnan.

## Related work

### Literature review

Rural revitalization is a central topic in contemporary academic research [19]. Several scholars have undertaken studies of rural revitalization in Hebei Province, China, using advanced techniques such as neural networks and spatial econometric models. The study has culminated in the development of a comprehensive system of evaluation indicators that assess the multifaceted nature of urban-rural functionality [20]. B&B industry plays a pivotal role in promoting the high quality of rural revitalization strategy and thus has received the attention of many scholars [21].

Scholars’ rural B&B industry research topics include B&B industry trend prediction, B&B development issues analysis of B&B industry management norms [22], such as rural B&B energy consumption prediction, rural B&B public finance burden assessment, the active role of rural B&B in tourism development, tourism poverty alleviation and rural revitalization, and the integration of rural B&B with other industries [23]. Few studies are based on machine learning to analyze the problems of B&B management, thus the research in this paper is innovative. Machine learning for textual information mining is an area of interest for researchers. The application of sentiment analysis to online review text has produced remarkable results in a variety of fields. Its integration with the latest online B&B review data provides objectivity and significant value, mitigating the resource-intensive nature and potential for large-scale error associated with traditional questionnaire-based approaches. [23] used the LDA approach to analyze the data of B&Bs. [24] studied the data related to Airbnb using LDA. [25] used traditional machine learning methods for Airbnb data in the Beijing area. Bert was applied as a newer and better text analysis model for various aspects [26]. [27] used Bert for drug name extraction, and some scholars used Bert model for language analysis [28]. The superiority of Bert model is widely proven, but it has not been applied to the analysis of B&B reviews from the perspective of rural revitalization. Sentiment analysis can be used to analyze the evolution of consumers’ purchase intention [29]. There are also many approaches to sentiment analysis; LSTM can be used to analyze online discussions [30], and CNNs have also been shown to be a model that can be used [31].

The majority of stakeholder theory research in tourism has focused on sustainable ecotourism [32], community tourism stakeholders [33], and tourism destination development planning [34]. In recent years, research on rural tourism stakeholders has become more focused, including the study of rural tourism value chains [35] and the exploration of governance models based on the rural edge theory [36].

The application of evolutionary games to rural tourism has also received attention from scholars. Research in hotel management has used vertical differentiation game models to determine optimal strategies when competing on hotel quality and price and to determine multi-win strategies for hotels when cooperating with third parties [37, 38]. Other studies have found that dynamic pricing planning and revenue growth are beneficial for the sustainable development of hotels [39]. Game theory has been used to analyze the complex network of relationships between the government, businesses, and residents in rural tourism, which can help understand the role of government [40]. Studies have also shown that interactions between operators within the same industry are simultaneous games, while those between industries are sequential games, and the behavior of participants changes over time [41]. Sequential game models have been used to explore the relationship between competition and complementarity among tourism destinations [42], and game theory has been used to analyze different tourism product forms to determine the best combination [43]. In contrast to classical game theory, which assumes perfect rationality, evolutionary game theory assumes limited rationality, which is more in line with real-world dynamics. As a result, evolutionary game theory is a valuable research tool [44]. This shift in assumption has attracted scholarly attention and led to a variety of applications. Some research has developed tripartite evolutionary game models to elucidate the evolution of government green development behavior in the field of construction waste recycling [17]. Others have explored the evolutionary mechanisms underlying green innovation behavior in construction enterprises [45]. In addition, the study of firms’ adoption of green development behaviors is a central facet of evolutionary research [46]. Furthermore, some studies have introduced novel and effective inventory solutions to address bankruptcy-related challenges [47]. The interplay between the evolutionary dynamics of participants in evolutionary games and their environmental context has also attracted academic interest [48]. Evolutionary game theory has also been applied to the analysis of complex networks involving governments, businesses, and residents in the context of rural tourism. This analytical framework helps to understand the role of government in such settings [40].

### Literature gap

Previous studies have extensively investigated problem perception and management within the B&B industry from a variety of perspectives, providing valuable scientific guidance for this paper. However, there are certain gaps in the existing research landscape due to different research perspectives. Much of the current research focused on theme extraction primarily employs traditional analytic methods and LDA machine learning techniques. This approach overlooks the superior performance potential of the Bert model, which has remained unexplored in the context of B&B review analysis. Furthermore, previous studies have predominantly relied on word frequency as an analytical metric, often neglecting the crucial aspect of reviewer sentiment. Conversely, some research has focused solely on sentiment analysis, with minimal attention to topic extraction. In particular, the application of evolutionary game methodology has yet to be explored in the context of Yunnan B&Bs, particularly in the domain of stakeholders involved in optimizing the management of Yunnan B&Bs. This represents a significant research gap in the existing literature.

To address these gaps, this paper presents two major improvements. First, we propose a novel topic extraction model and a highly accurate sentiment analysis model, which form the basis of a machine learning framework for accurate analysis of Yunnan B&B review data. This framework is designed to uncover the most critical issues and suggest directions for optimizing Yunnan B&B management. Second, we identify the evolutionary game participants in the Yunnan B&B management optimization process based on the results derived from the machine learning framework. The main stakeholders include the government, B&B operators, and tourists. We construct a payment matrix and establish a replicator dynamic equation to conduct an in-depth analysis of the asymptotic stability of each stakeholder and the evolutionary stability strategies under different conditions. To further enrich our findings, numerical simulations are performed to identify crucial parameters that influence the evolutionary outcomes and trajectories. This paper provides valuable management insights for optimizing the management of B&Bs in Yunnan, with the overall aim of providing a reference and support for the development of Yunnan’s B&Bs and the broader cause of rural revitalization.

## Theoretical foundations of machine learning tools

### Bert model

**Fig 1:**
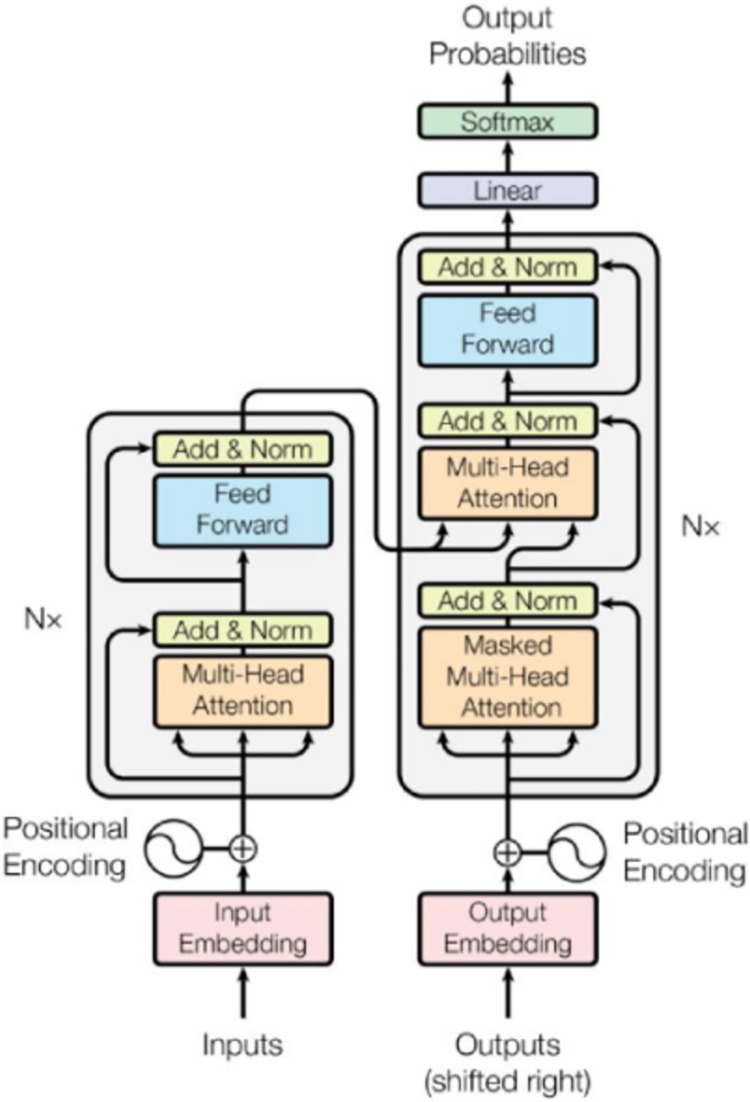
Transformer model structure.

The Transformer model is a model proposed for textual information processing [49], using an attention mechanism, which has achieved excellent results in the field of natural language processing, especially in textual information mining processing. Its model architecture is shown in Fig 1 [50]. The main architecture consists of multiple encoders and multiple decoders.

The Bert model is a variant form of the Transformer model [40], which has achieved very good performance since it was proposed, and it uses a dual-transformer structure that can utilize information from both directions.

The Bert model introduces the sine and cosine functions to encode the position with the following equations.


$$PE_{\left(pos,2i\right)}=sin\left(\frac{pos}{10000^{\frac{2i}{d_{model}}}}\right),PE_{\left(pos,2i+1\right)}=cos\left(\frac{pos}{10000^{\frac{2i}{d_{model}}}}\right)$$ (1)

Where, pos indicates the position of the word and denotes the dimension of the word vector, which is the dimensional ordinal number of the word vector. According to the nature of the trigonometric function, a certain dimension of the vector or all can represent the combination of position vectors, thus the Bert structure can use not only absolute position information but also relative position information.

The Bert model uses a multi-headed self-attentive mechanism to enhance the accuracy of the model for text feature mining. The principle is as follows: the model takes the word vector matrix of the text and constructs three matrices by linear variation, representing query (Query, Q), key value (Key, K), and value (Value, V), respectively. The attention mechanism requires an attention score, which is calculated as follows.

$$Attention(Q,K,V)=softmax\left(\frac{QK^{T}}{\sqrt{d_{k}}}\right)V$$ (2)

where QK^T^ is the self-similarity between Q and K computed using the inner product, and d_k_ is the dimensionality of the row vectors in Q and K. The calculation ensures that each element of the result can include the information of all words. And a single-word head does not extract all the word information well, thus the model introduces a multi-head self-attentiveness mechanism, which is calculated as shown below.

$$MultiHead(Q,K,V)=Concat(head_{1},head_{2},\ldots ,head_{k})W^{o}$$ (3)

where head_i_ = Attention(Q_i_, K_i_, V_i_).

### Convolutional neural networks (CNNs)

Convolution Neural Networks (CNNs) is a variation on ANNs [51] that use convolution to extract features. CNNs have been used with good results since they began to be used [49] and are currently the more popular neural network [52].

**Fig 2:**
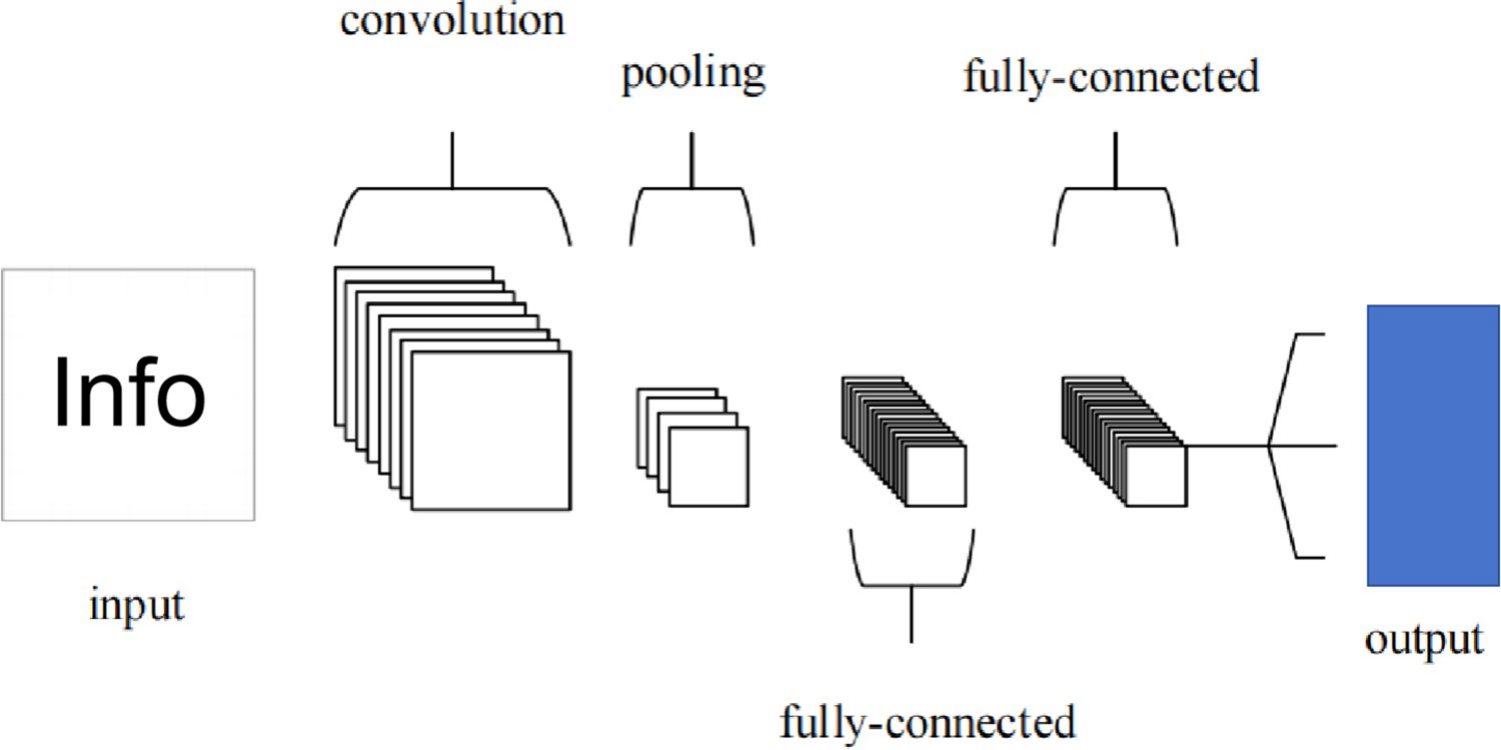
An simple CNN architecture.

The CNN is divided into three main layers: the convolutional layer—the main role is to extract features; the max pooling layer—the main role is to downsample without damaging the recognition results, and the fully connected layer—the main role is to classify. This is shown in the Fig 2.

### LSTM and GRU

**Fig 3:**
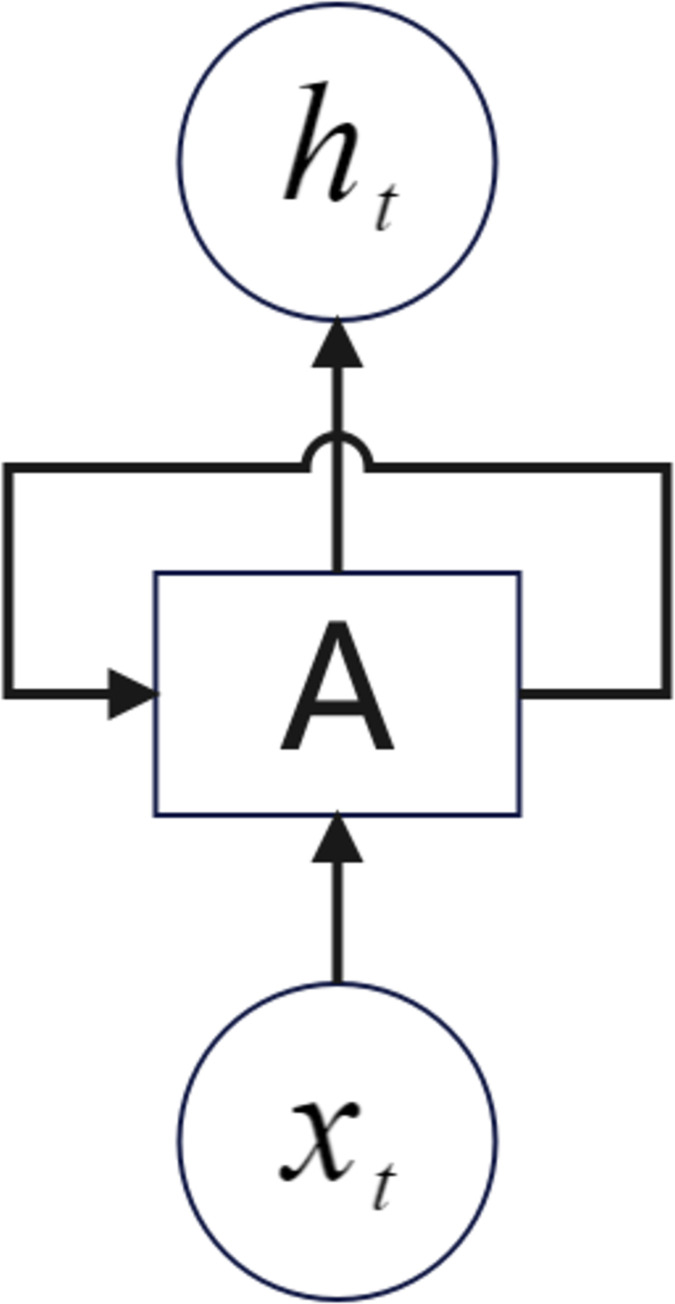
RNN model. Where x_t_ is the input information at moment t and h_t_ is the input information at moment t. Neuron A can recursively call itself and pass the information at moment t-1 to moment t.

LSTM (Long Short Term Memory) is an optimized form of RNN, which was proposed [53] and improved [54] to perform well in the work of processing sequential data, especially when there is a connection between before and after data. The RNN structure is shown in Fig 3 [55]. The GRU model is an optimization of the LSTM model [56], and this paper uses both The LSTM is an optimized form of RNN, which solves the problem of long and short-term memory between front and back, but the LSTM has too many parameters, which affects the computational efficiency and requires huge computational resources, thus GRU optimizes the LSTM by optimizing the number of parameters and thus the LSTM.

### Performance evaluation

For the model of sentiment analysis, we use the [57, 58] approach for measurement, using a confusion matrix, to calculate the F1 score. For the effect of active learning, we measure the model effect of active learning by comparing the accuracy and F1 scores. In general, the accuracy and F1 scores decrease after using active learning, but if the decrease is in an acceptable range, it shows that active learning is helpful.

Classifier accuracy is measured with the help of a confusion matrix. This matrix provides the number of right and wrong predictions by comparing actual target values. It consists of four parameters: (1) true positive (TP): shows how many actual true values the model predicted as true; (2) true negative (TN): shows how many actual false values the model predicted as false; (3) false positive (FP): shows how many actual false values are predicted as true; and (4) false negative (FN): shows how many actual true values are predicted as false.


$$Accuracy=\frac{TP+TN}{TP+TN+FP+FN}$$ (4)

$$Precision=\frac{TP}{TP+FP}$$ (5)

$$Recall=\frac{TP}{TP+FN}$$ (6)

$$F1score=2*\frac{Precision*Recall}{Precision+Recall}$$ (7)

## Data processing, analytical model and method parameter setting

### Perceptual and analytical framework

**Fig 4:**
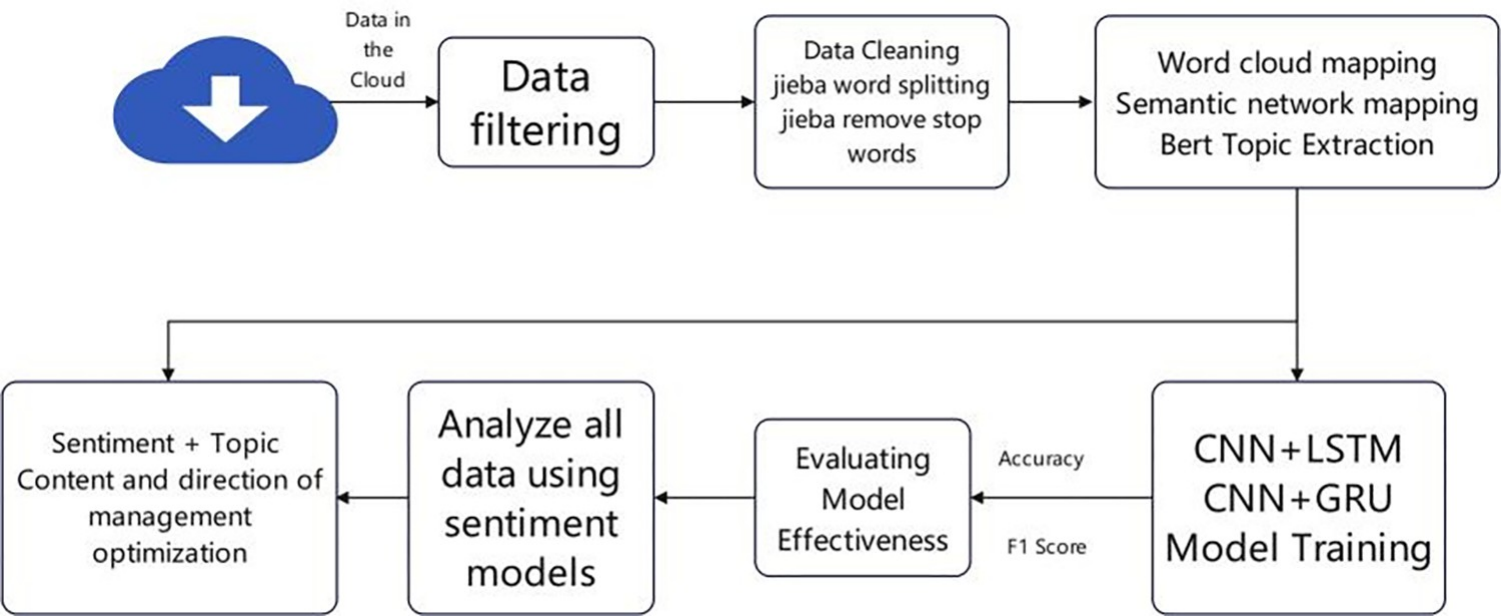
A perceptual and analytical framework for optimizing the content and direction of B&B management in Yunnan.

The analysis-awareness model proposed in this paper is shown in Fig 4. Subject terms can help us extract key information from a huge amount of data, but this information is neutral and not helpful to improve management. Firstly, we select suitable data from the Internet and then perform data cleaning and data pre-processing. Data pre-processing includes irrelevant character filtering, text splitting, and deactivation of words. The word separation process of comment data is a necessary step before applying Bert topic model and machine learning. Because the comment content contains a large number of expressions, URLs, punctuation and hashtag, @, and other symbols, the irrelevant characters in the comment content should be filtered before the word separation process. In this paper, the text content is extracted from the original dataset by regular expressions, and then the text data is processed by Jieba word splitter, which is the best Chinese word splitter in Python language system, and its main principle is to determine the association probability between Chinese words by using Chinese lexicon, and then produce the result with 97% accuracy. There are many deactivated words in the post-sorting data. To reduce the interference of topic irrelevant words, this paper removes the meaningless words in the text according to the deactivated word list and then draws word cloud and semantic relationship graphs.

### Analytical model and method parameter setting

**Fig 5:**
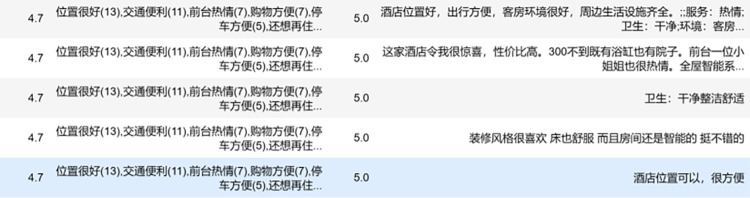
Visitor reviews raw data display.

**Fig 6:**
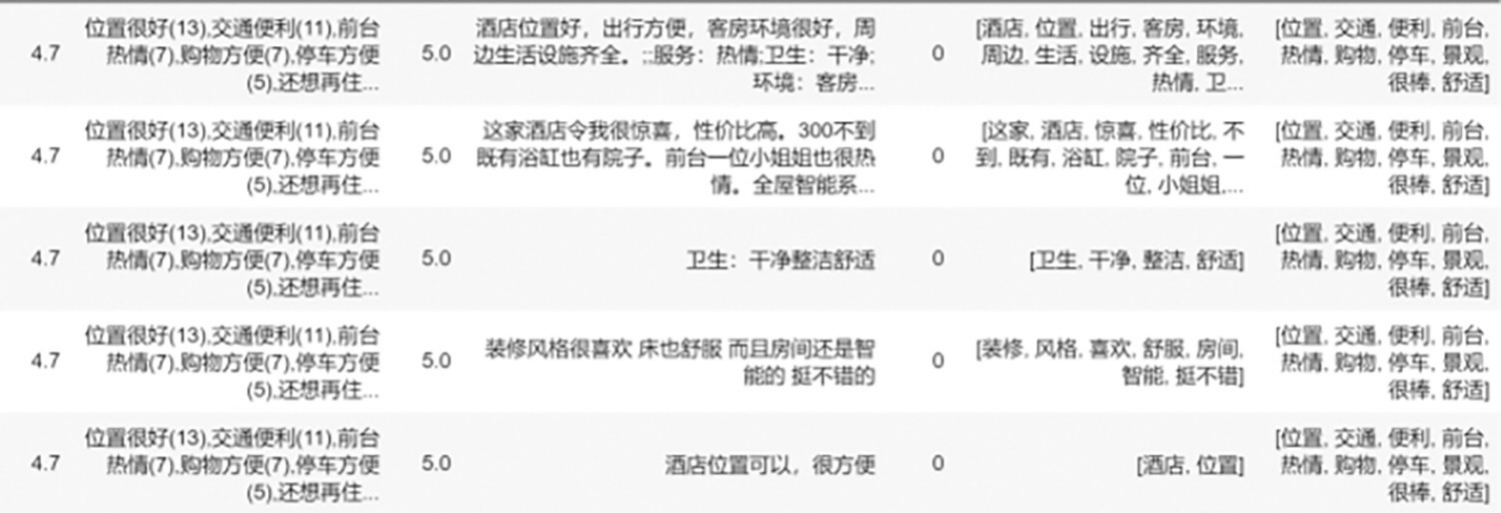
Display of review data after data pre-processing.

This paper crawls the evaluation data of Yunnan B&Bs on Ctrip.com through web crawler technology. Firstly, based on the previously existing literature, several scholars crawled data research for Ctrip.com, which proves that Ctrip.com has certain representativeness. Moreover, Ctrip.com has a clear classification of B&Bs and has an exact B&B classification function. The data collection process took place at the end of December 2022 and involved the retrieval of textual content from visitor reviews of the prominent B&Bs operating during this period. For each B&B, approximately 20 data entries were retained and organized based on the respective travel website. The crawler obtained 65535 pieces of data, as shown in Fig 5. Among them, the overall rating is the comprehensive rating of B&B calculated by Ctrip.com according to the website’s calculation method, the label is the evaluation label derived from the website and the guests, which shows the guests’ evaluation of B&B in a broad sense, the guest rating is the rating derived from the guests’ scoring and the website’s calculation, and the review content is the textual evaluation of B&B by the lodging guests. After filtering out the missing and invalid data, we obtained valid data bars and pre-processed the data, the results are shown in Fig 6, except for the content shown above, the label in the figure is the sentiment rating, 0 represents positive sentiment, 1 represents negative sentiment, "review_content_cleaning" and "label_cleaning" is the cleaning of the label and review. Cleaning" is the result of word separation of label and comment content. The data is pre-processed to obtain the word cloud map after data pre-processing. In this paper, we use the WordCloud library for word cloud mapping, which generates a word cloud map from the words after data cleaning by the WordCloud function. In this paper, ROST CM 6 (ROST Content Mining System Version 6.0) is introduced to perform "social network and semantic network analysis" on the comment text to obtain the semantic relationship graph.

In the second part, the Bert model is used to analyze the tags (directly generated by the travel website, such as "good location, convenient transportation, friendly front desk, convenient shopping, convenient parking") and the content of the reviews for topic word analysis. It has many advantages, especially for Chinese language processing. Because Chinese does not have tense-variant forms of verbs, nouns, and adjectives, location information is particularly important, and the Bert model can make good use of location information. The traditional bag-of-words model does not consider the association between words, and other machine learning algorithms are relatively lacking in the use of word position, and are prone to gradient explosion and gradient disappearance problems. The traditional fully connected neural network is independent of each other from layer to layer and from node to node, resulting in the current sequence of inputs being independent of the previous sequence of inputs, which does not meet the requirements of textual information, while the multi-headed attention mechanism introduced by the Bert model can handle textual information better.

In this paper, we selected 4 as the number of topics for topic word analysis and obtained "topic-word distribution", topic intensity distribution, topic distance map, and topic hierarchy clustering map by Bert model.

**Fig 7:**
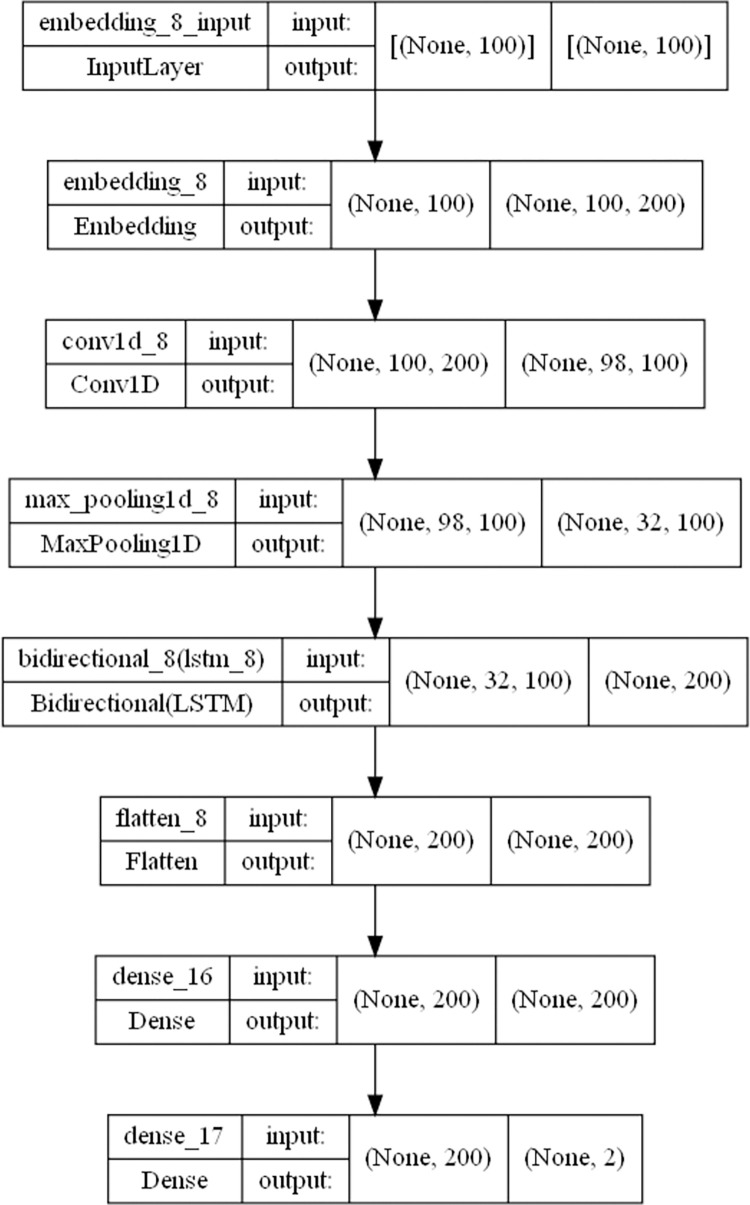
Deep learning network structure diagram of CNN+BiLSTM network.

**Fig 8:**
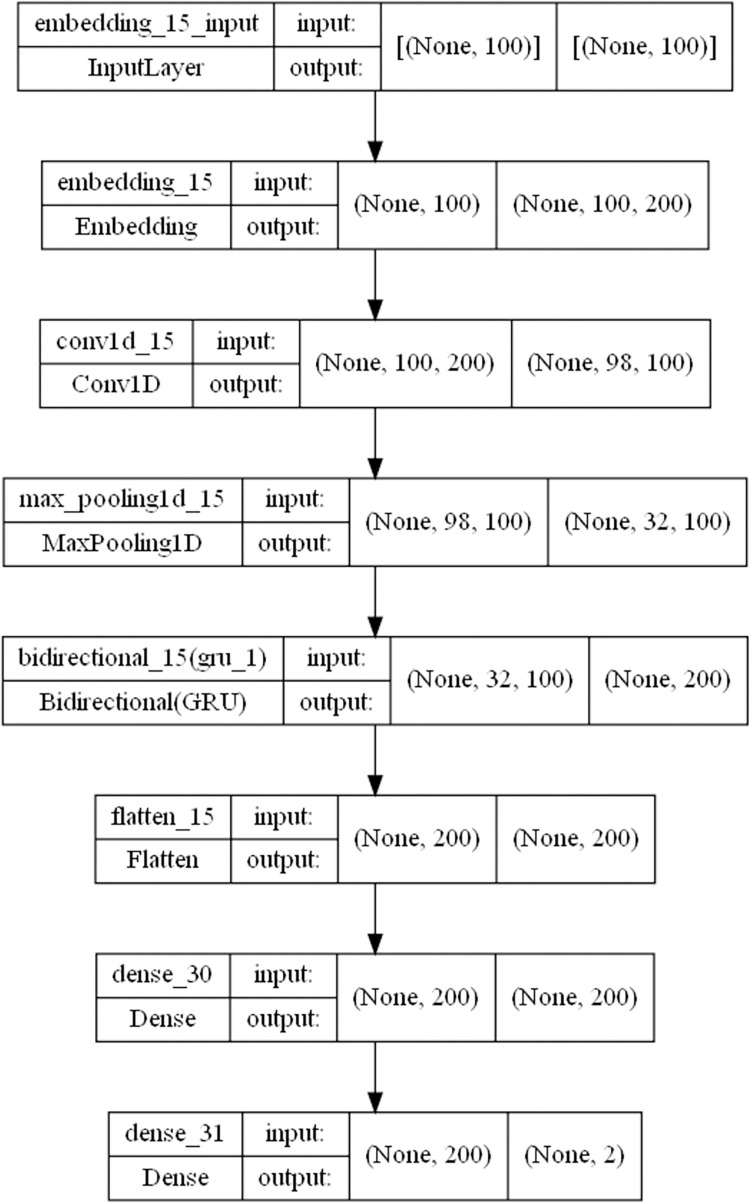
Deep learning network structure diagram of CNN+BiGRU network.

The third part is the analysis of sentiment by machine learning model. In this paper, we use CNN+BiLSTM to build a model for emotion recognition, the main idea is to use CNN for feature extraction and BiLSTM for prediction analysis, the specific model structure is shown in Fig 7. The first layer is the input layer; the second layer is the embedding layer, which transforms the index of the input layer into a word vector; the third layer is the convolutional layer, which extracts features of different dimensions using convolutional kernels; the fourth layer is the maximum pooling layer, which extracts core features; the fifth layer is the BiLSTM layer; next is the fully connected layer and the output layer, which maps the information as the output of the model. Because deep learning is supervised learning and requires labels, we label the data with positive and negative sentiments. The labeled data is then divided into a training set and a test set for training. As a reference, this paper uses both CNN+BiGRU models for sentiment recognition, as shown in Fig 8, and the structure is similar to the CNN+BiLSTM structure shown above, except that the BiLSTM layer is replaced with a BiGRU layer. Finally, the sentiment classification of all data is evaluated using the model to obtain the sentiment distribution map.

Specifically, this paper uses the deep learning tool Tensorflow for model building. Dividing 70% of the data into the training set and 30% into the test set, there are 28229 training data and 12735 test data. The maximum length of the text is set to 100, the activation function is set to "relu", the loss function is set to Cross Entropy, and the optimizer is set to "Adam".

## Analysis of the study results

### Analysis of the content for all reviews

#### Word cloud graph

**Fig 9:**
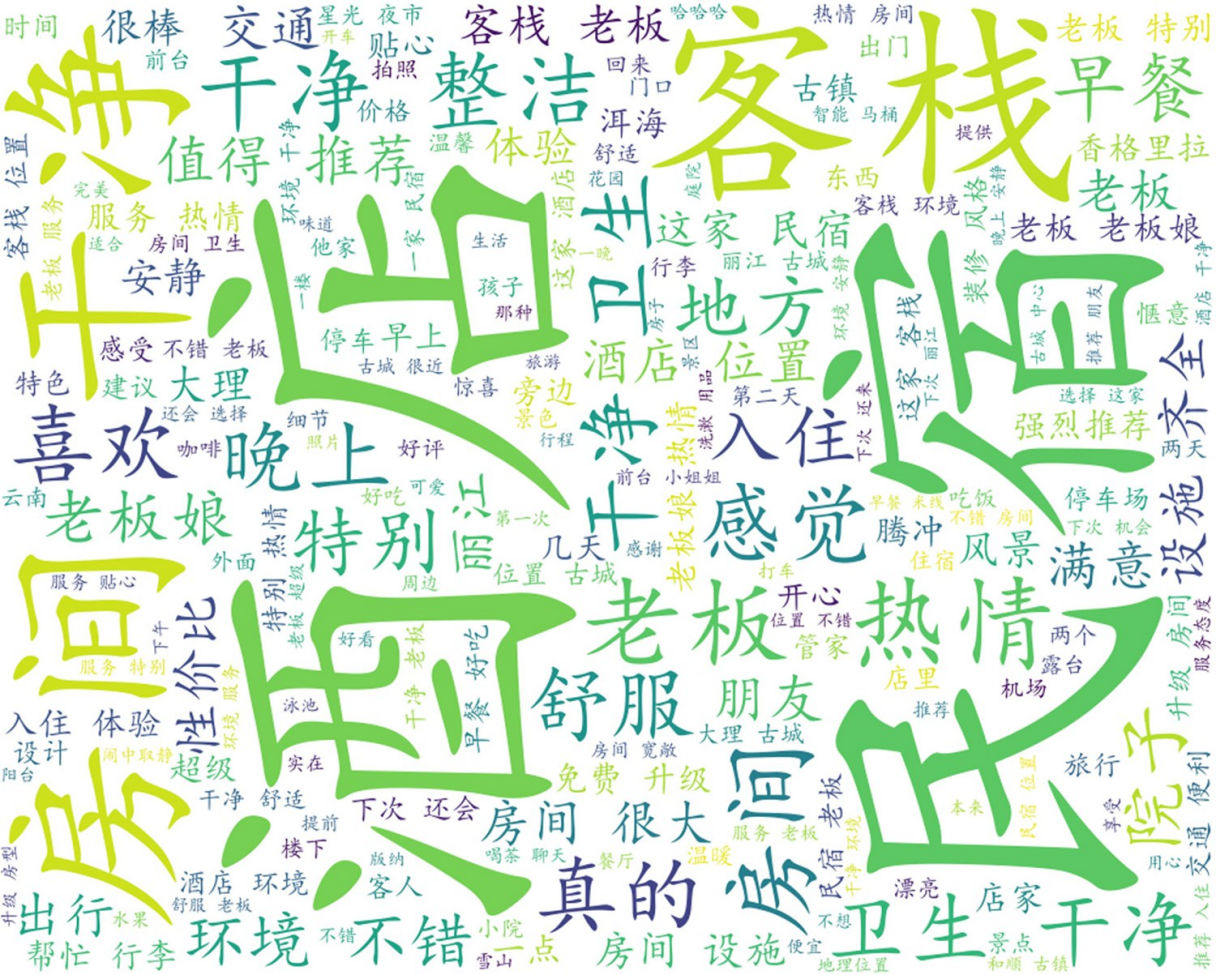
All reviews generate word cloud graph.

**Fig 10:**
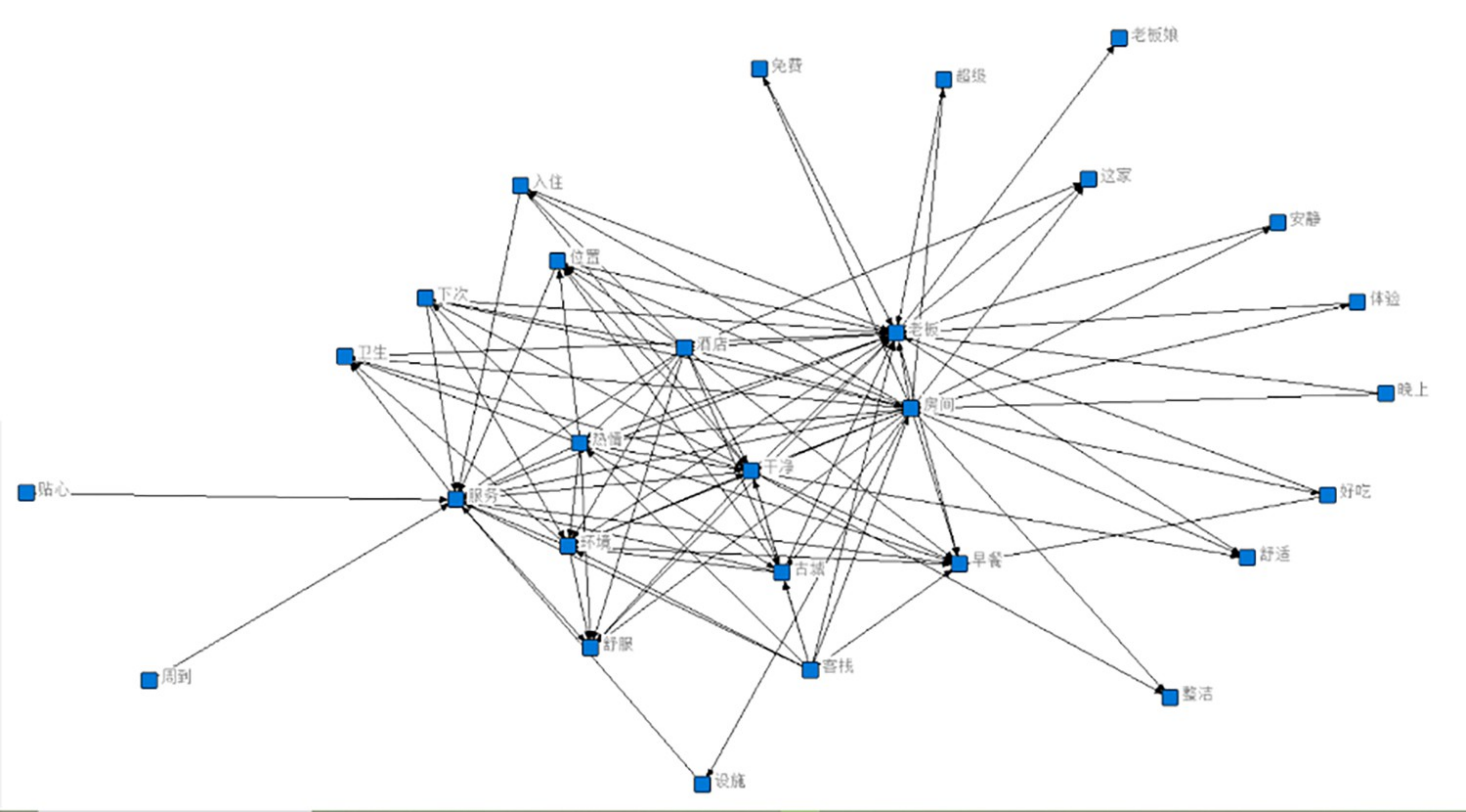
Semantic network graph of all reviews.

In this paper, we first generate word cloud graphs (shown in Fig 9) and semantic network graphs (shown in Fig 10) based on the comment content, and the process of generating semantic network graphs yields and co-occurrence matrix word list, and here the first nine items are selected for analysis based on word frequency ordering (shown in Table 1).

**Table 1:** Co-term matrix of main high-frequency words.

	Boss	Room	Hotel	Service	Clean	Hospitality	Environment	Guest House	Ancient City
Boss		8558	4561	4727	6331	7716	4996	4619	3963
Room	8558		5174	5715	7997	6037	4424	4071	3840
Hotel	4561	5174		4015	3475	3280	2909		2090
Service	4727	5715	4015		4434	4129	4573	2382	2212
Clean	6331	7997	3475	4434		4578	4078	2866	2778
Hospitality	7716	6037	3280	4129	4578		3585	3039	2696
Environment	4996	4424	2909	4573	4078	3585		2109	2042
Guest House	4619	4071		2382	2866	3039	2109		2328
Ancient City	3963	3840	2090	2212	2778	2696	2042	2328	

The word cloud diagram shows the most important parts of the guests’ evaluation. Apart from the two specific terms "hotel" and "B&B", the word cloud map is mainly characterized by two aspects, namely the hardware environment and the emotional experience. The words "room", "clean", "hygiene" and "facilities" appear more frequently, reflecting the fact that guests care about the hardware and facilities of their stay, which is natural. At the same time, words such as boss, boss lady, warm, attentive, and helpful are also more frequent in the word cloud graph. This shows that for B&B guests, the emotional experience is a very important part of the experience, greatly influencing the guest’s living experience and thus the rating and revenue of the B&B.

Through the co-word matrix, it can be concluded that the terms boss, room, service, clean, and passion appear more frequently, and the mutual linkage relationship is derived after statistical analysis. The frequency between owner and room is 8558 times, between the room and clean is 7997 times, and between owner and enthusiasm is 7716 times. In the semantic network, "owner", "enthusiasm" and "room" are the most closely related to other words and have the highest frequency of co-occurrence, so they are the core words in the whole semantic network. The three feature words make the whole semantic network interconnected. From this, we can conclude that the guests’ requirement for the hardware condition of the room is low, just clean, while they care more about the enthusiasm of the owner, i.e. the emotional experience.

From the semantic network diagram, it is concluded that the boss is the most important word in the whole guest evaluation and is the absolute core word, while the next level core words are environment, cleanliness, and warmth, which can be corroborated with the results derived from the word cloud diagram and co-word matrix diagram.

In summary, guests’ requirements for B&Bs focus on objective physical conditions and emotional experiences. The objective material conditions include the cleanliness and tidiness of the B&B rooms, the environment of the B&B, and the location of the B&B, etc. Among them, the guests consider the most important thing to be the cleanliness and tidiness of the rooms. Among them, guests consider the cleanliness of the B&B rooms to be the most important, and cleanliness accounts for the majority of the guest evaluation indicators, while the environment, location, and other facilities are not important. The word "owner" was the absolute high-frequency word in the analysis, while other high-frequency words such as service, enthusiasm, help, and attentiveness were also highly related to the owner. This suggests that the owner and the attitude he or she displays is a more important factors in the management of a B&B than the hardware and facilities of the room. Whether it is the cleanliness of the room or the warmth and thoughtfulness felt by the guests, it is the owner’s business strategy. With warmth and thoughtfulness being more important, the most effective way to improve the quality of the B&B and its positive reviews is to improve the emotional experience of the guests. In this paper, based on the analysis of the word cloud diagram, it is concluded that the guests’ expectations and requirements for B&B, i.e. the requirement of clean rooms is sufficient, while the owner’s enthusiasm, consideration and help as the guests’ expectations are the parts that B&B operators should perceive and handle properly, and the results of which directly affect the income of B&B and the development of rural revitalization business.

#### Bert topic word result analysis

According to Bert’s algorithm, we derived four topic terms and related weights for the evaluation of Yunnan B&Bs, as shown in Table 2. Because the computer algorithm counts from "zero", the topic terms are numbered from "zero" in this paper. The four topics are summarized as general feeling, important characteristics of B&B, special characteristics of B&B, and degree of B&B evaluation expression.

**Table 2:** “Topic-word” distribution and their weights of all reviews.

topic 0	weights	topic 1	weights	topic 2	weights	topic 3	weights
Nice	0.43927944	Clean	0.24849837	Guest House	0.24554023	Owner	0.11199390
Environment	0.28423292	Enthusiastic	0.19112517	Dali	0.12043898	Really	0.06049972
Service	0.15229294	Environment	0.16449085	Owner	0.07561045	Owner’s wife	0.04099020
Service attitude	0.13828285	Comfortable	0.13264384	Ancient Town	0.06128995	Like	0.03804516
Owner	0.10956720	Recommend	0.13092234	Ancient Town	0.05980637	enthusiastic	0.03617461
Next time	0.10939759	Owner	0.12251722	enthusiastic	0.04198271	Special	0.03540516
Value for money	0.09788247	worth	0.10411297	This house	0.03317157	B&B	0.03427280
Enthusiasm	0.08544138	Beautiful environment	0.10268118	Characteristic	0.03315261	Next time	0.03375203
Hotel	0.07620009	Nice	0.09515807	Special	0.03239971	Super	0.02889560
Location	0.06211298	neat and tidy	0.09178416	Location	0.03144125	Room	0.02825806

Topic word 0 is overall feeling. This topic reveals the overall feeling of the guest for the B&B, words such as nice, environment, and warm are describing the overall feeling of the first impression. As a tourist, the first stop at the travel destination is the B&B, and the first impressions left by the B&B will greatly affect the guests’ feelings about the B&B, the guests’ satisfaction with the B&B, and then the guests’ subjective feelings about the location, affecting the travel mood, and to some extent, the travel experience, affecting the desire to spend. Thus, the management of B&Bs should focus on the first impressions they form for tourists.

Topic word 1 is an important characteristic of B&Bs. Words such as clean, welcoming, comfortable, environment, and owner are important characteristics for guests’ evaluation of B&Bs, and they are also what guests are most concerned about when staying in a B&B. The words with greater weighting mainly reveal the two elements that guests value most for B&Bs, infrastructure, and emotional experience: i.e., the requirement for clean and comfortable rooms and the requirement for warm treatment. From the perspective of rural revitalization, as villages with tourism resources, the folk style is relatively simple, the public facilities are relatively imperfect, and the information on tourism strategies is relatively lacking. On the one hand, besides the beautiful natural scenery, the local folk culture and humanistic style are also what travelers expect to experience, and the attitude of B&B operators is also a reflection of this; on the other hand, the enthusiastic B&B owners themselves give a good mood, and the travel tips provided by them will also give great help to travelers and improve the travel experience.

Topic word 2 is the special characteristics of the B&B. This topic reveals the special characteristics displayed by B&Bs, such as the location in ancient towns or old cities, or the existence of special and particular features of B&Bs themselves. In regions that rely on tourism to achieve rural revitalization, tourism generally accounts for a heavy economic boost, and travelers’ consumption is an important way to generate lo-cal income, so some B&Bs will adopt a differentiation strategy, i.e., by creating B&Bs with special features and characteristics to attract tourists.

The topic word 3 is the degree of evaluation of B&Bs. The words "really", "like" and "super" express the degree of evaluation and emotion of tourists. Although the word "super" does not directly indicate the management direction and has no practical meaning, it expresses the visitors’ emotions. Whether it is a positive or negative emotion will be further analyzed in a later study.

Of particular interest is the fact that the terms "boss" and "warm" appear in all four topics, indicating their importance in tourists’ evaluations. The most important thing that tourists value in a B&B is the owner and his or her attitude.

**Fig 11:**
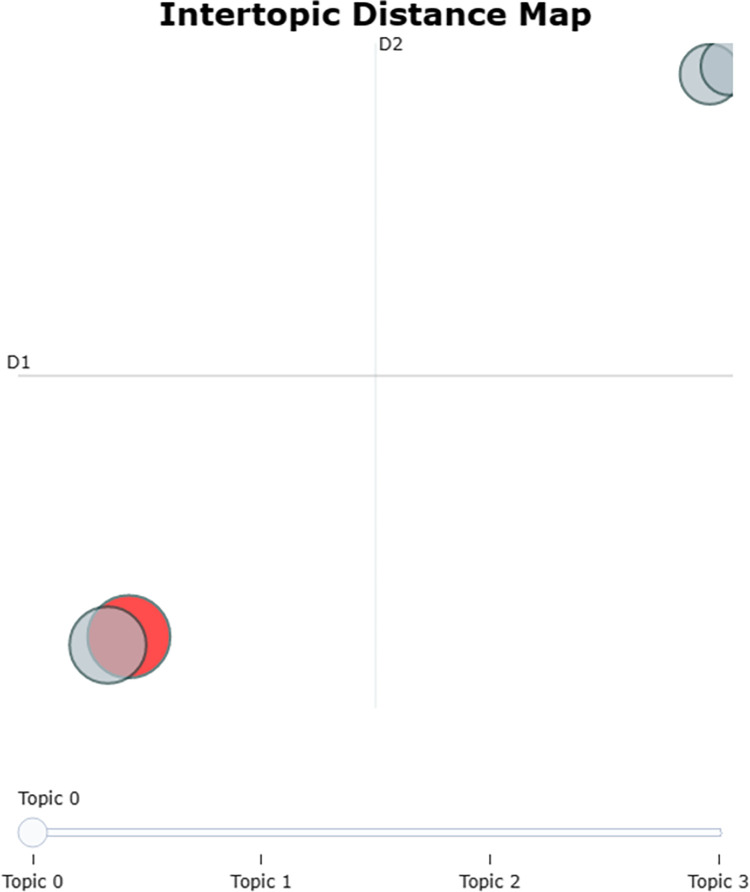
Topic spacing graph.

**Fig 12:**
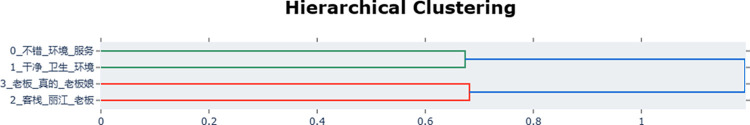
Topic hierarchy clustering graph.

Fig 11 shows the topic spacing graph, in which the two circles in the lower left corner represent topic 0 and topic 1, and the two circles in the upper right corner rep-resent topic 2 and topic 3. Fig 12 shows the topic hierarchy clustering graph, in which topic 0 and topic 1 are clustered into the same class, and topic 2 and topic 3 are clustered into the same class. Both the topic spacing diagram and the topic hierarchy clustering diagram give the same results, i.e., the four topics derived from the topic ex-traction in this paper are classified into two categories, topic 0 and topic 1 are similar topics and differ from the other category (topic 2 and topic 3).

**Fig 13:**
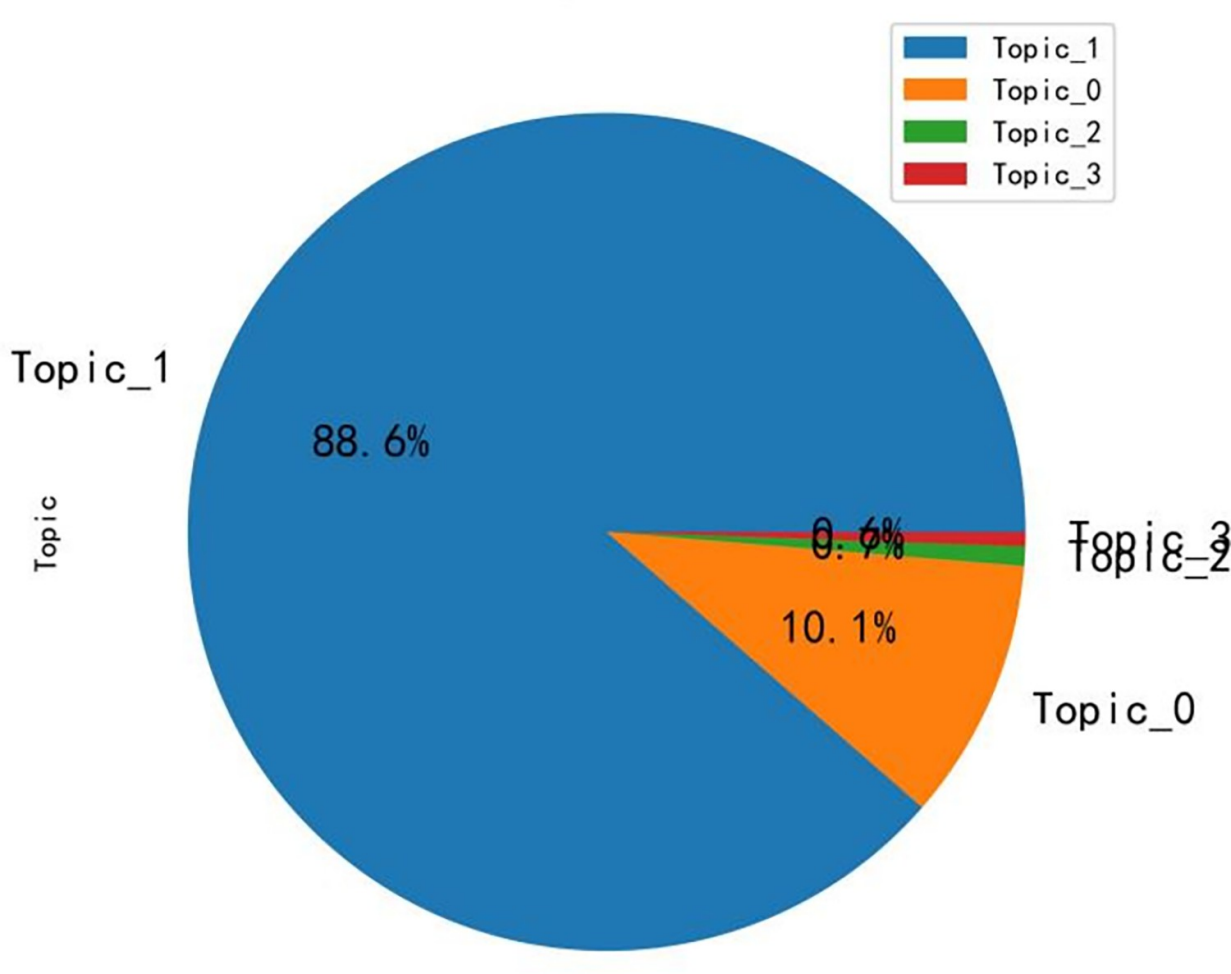
Topic word intensity distribution graph.

According to the matching relationship between topics and comment contents, the distribution of topic word intensity is obtained, as shown in Fig 13. The figure yields that topic 1 has the highest percentage of 88.6%, topic 0 has 10.1%, topic 2 has 0.7%, and topic 3 has 0.6%. Combining the clustering results, it can be concluded that the first category (topic 0 and topic 1) accounted for 98.7%, thus concluding that topic 0 and topic 1 are the information that local governments and B&B operators need to focus on planning for optimizing B&B management based on the perspective of rural revitalization.

#### Sentiment analysis model results analysis

The Bert topic model mines topic words for visitor reviews, and the topic words reveal the direction to focus on in the management of this matter. However, the topic words can only mine the keywords to reveal the main areas and scope of the imposed management; the mined topic words are neutral and cannot derive the emotional color of the visitors, thus, the sentiment analysis is very important for the B&B evaluation.

**Fig 14:**
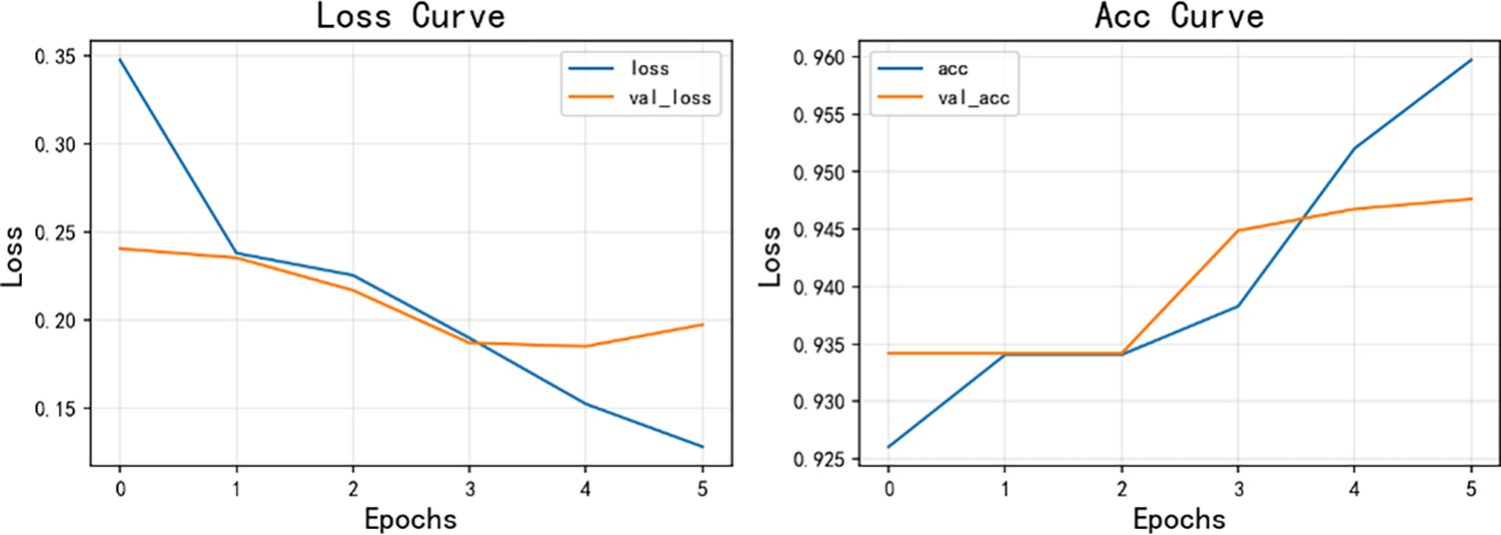
CNN+BiLSTM model training curve.

**Fig 15:**
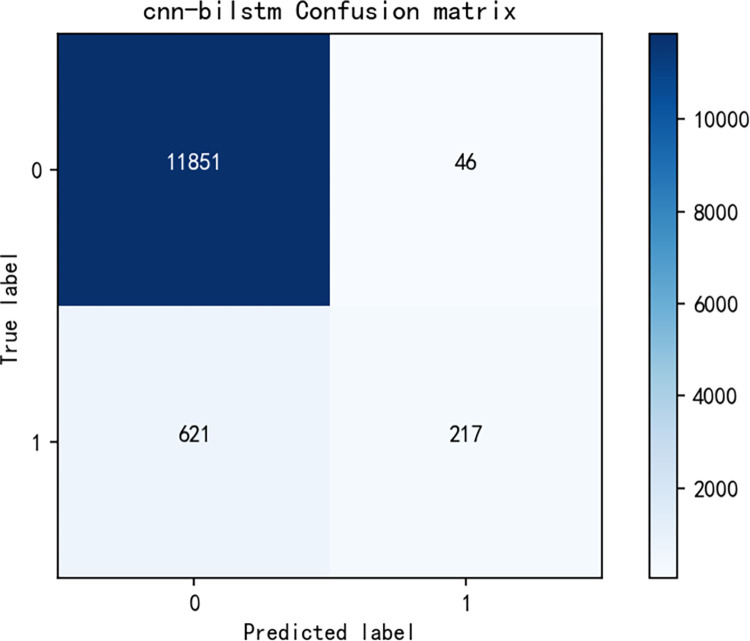
Confusion matrix for CNN+BiLSTM model results.

**Fig 16:**
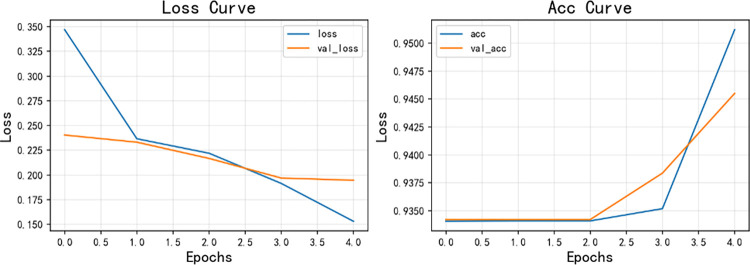
CNN+BiGRU model training curve.

**Fig 17:**
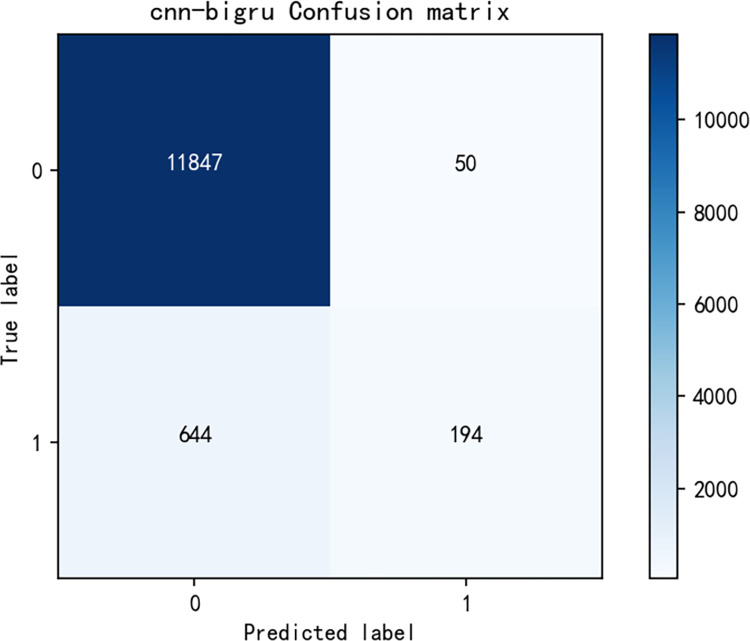
Confusion matrix for CNN+BiGRU model results.

Fig 14 shows the model training curve of CNN+BiLSTM, from which it can be concluded that the model training is successful, and the accuracy curve and model loss curve converge successfully. Fig 15 is a graph of the confusion matrix results for model evaluation. The model was evaluated on the test set, and the model accuracy reached 0.9476 with an F1 score of 0.9346, and all data are higher than the commonly used sentiment analysis models, thus, the model performance of the sentiment analysis model used in this paper is excellent on the data in the research area of this paper. Similarly, Fig 16 shows the model training curve of CNN+BiGRU, and Fig 17 shows the confusion matrix. The model accuracy reaches 0.9455 and the F1 score is 0.9312. The BiGRU model and the BiLSTM model work similarly because the BiGRU model requires fewer parameters and is therefore a better choice. The machine learning model used in this paper is compared with other machine learning algorithms mentioned. The comparison is shown in Table 3.

**Table 3:** Comparison of the effects of machine learning models.

	Accuracy	F1 score
CNN	0.8912	0.8868
LSTM	0.9257	0.9123
GRU	0.9213	0.9015
CNN+BiLSTM	0.9476	0.9346
CNN+BiGRU	0.9455	0.9312

**Fig 18:**
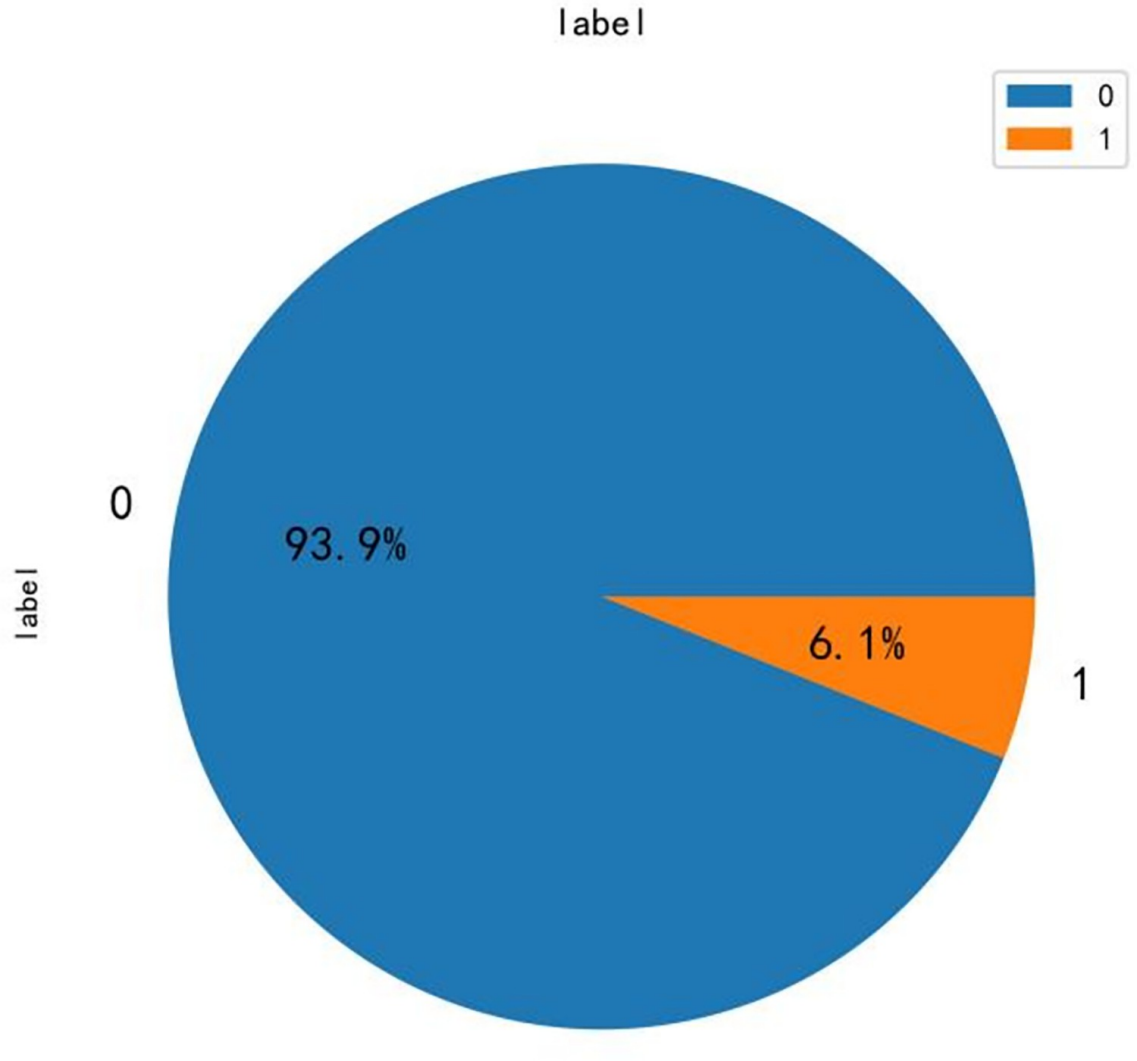
Sentiment analysis of all reviews as a percentage graph, where 0 is positive sentiment and 1 is negative sentiment.

The performance of the models is excellent, and thus the machine learning model for analyzing the sentiment of B&Bs is successfully constructed in this paper and reaches a high level of similar models. The results of using the model to analyze the sentiment of B&B are shown in Fig 18.

From Fig 18, it can be concluded that 93.9% of the data analyzed in this paper are positive emotions. The positive sentiment is heavier, but in reality, the B&B does not meet the expectations and requirements of tourists. Similarly, shopping reviews on the website are overwhelmingly positive, but the actual user experience of the product is not as good as it could be. Therefore, to further explore the management improvement direction of Yunnan B&Bs, this paper extracts topics for negative sentiment evaluations.

### Negative sentiment review topic extraction

To further explore the direction of B&B management optimization and the scope to be focused on, this paper conducts topic word extraction for negative sentiment reviews to clarify the reasons for the dissatisfaction of tourists who give bad reviews. The sentiment analysis model targets all reviews and aims to classify positive sentiment and negative sentiment, so it is meaningless to train the sentiment analysis model again, so the sentiment analysis model is not trained and evaluated for negative sentiment data.

**Fig 19:**
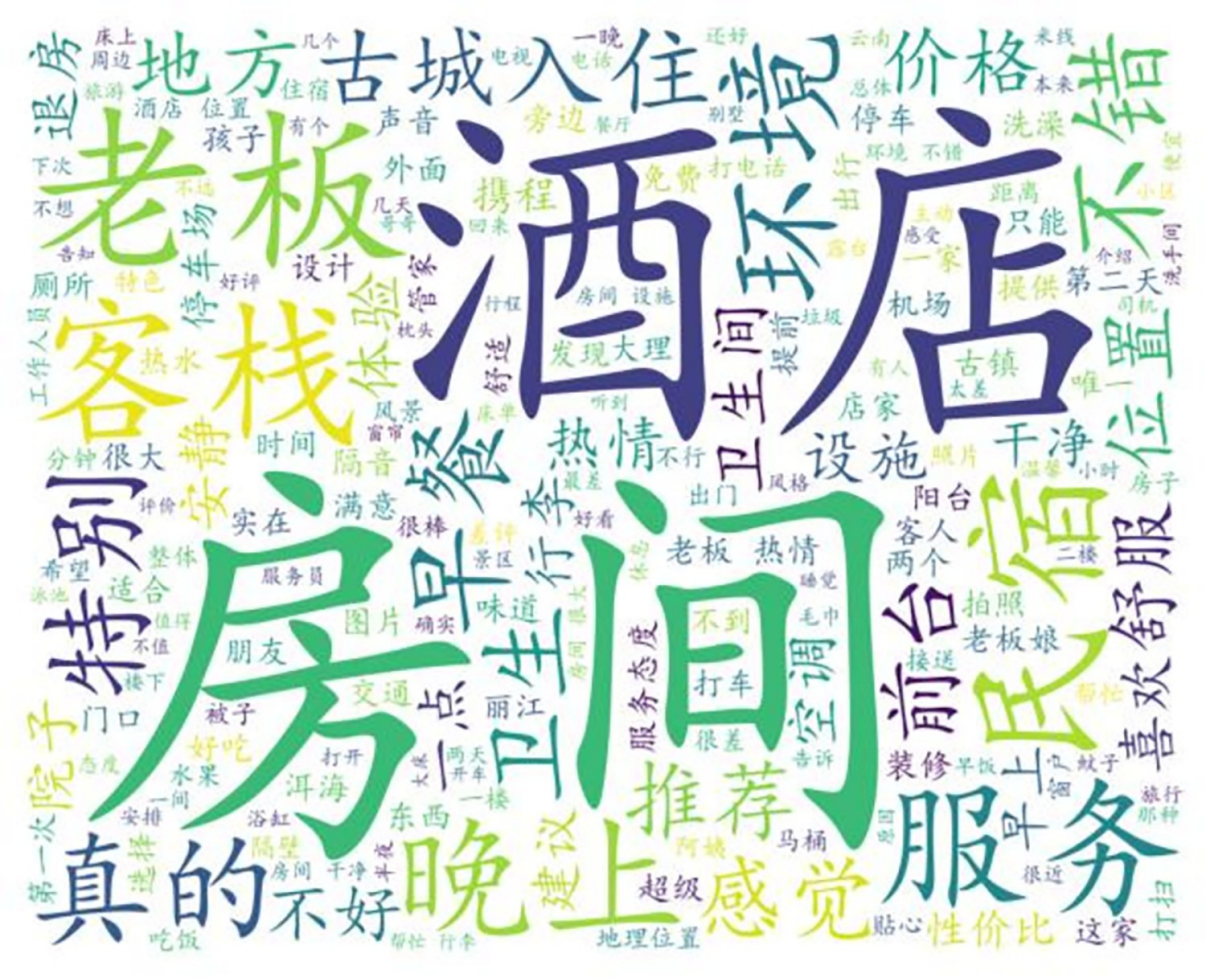
Negative sentiment review word cloud graph.

**Fig 20:**
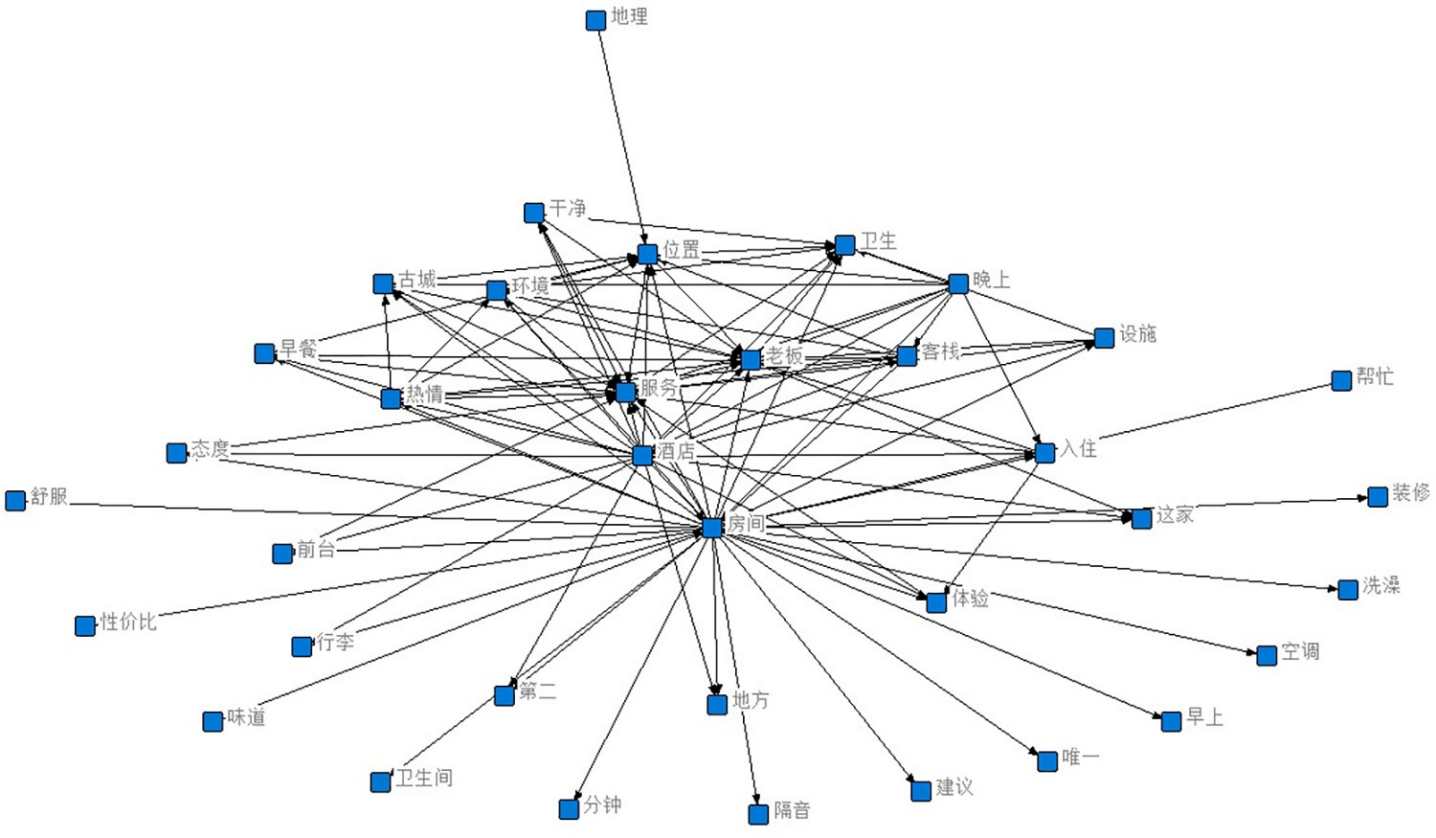
Semantic network graph of negative sentiment reviews.

After the model screening, 2803 negative sentiment data were obtained. The word cloud map (Fig 19), the co-word matrix table of high-word frequency words (Table 4), and the semantic network map (Fig 20) were derived from the experiments.

**Table 4:** Matrix table of high word frequency co-words for negative sentiment reviews.

	Room	Hotel	Owner	Service	Guest House	Environment	Location	Clean	Night
Room		444	390	361	236	233	270	239	283
Hotel	444		235	269	139	152	201	159	204
Owner	390	235		185	201	155	179	129	168
Service	361	269	185		123	164	144	156	145
Guest House	236	139	201	123		100	125		100
Environment	233	152	155	164	100		112	121	
Location	270	201	179	144	125	112		98	118
Clean	239	159	129	156		121	98		103
Night	283	204	168	145	100		118	103	

According to the word cloud diagram, it can be concluded that in addition to generic words such as room and hotel, words such as service, boss, night, enthusiasm, and bad take up a larger proportion. It indicates that the main reasons for tourists’ dissatisfaction are bad service, lack of enthusiasm, and poor behavior of the boss, and the secondary reasons are objective B&B conditions such as bad location and not quiet enough at night.

Through the co-word matrix, it can be concluded that the terms boss, service, environment, and evening appear more frequently, and the statistical analysis has led to the mutual linkage relationship. The frequency between owner and room is 390 times, between room and clean is 239 times, and between room and service is 269 times. In the semantic network, "owner", "enthusiasm" and "room" are most closely related to other words and have the highest frequency of co-occurrences, so it can be concluded that the guests’ requirements for the hardware condition of the room are low, and only the cleanliness is important. It can be concluded that guests have lower requirements for the hardware condition of the room, which is only clean; and they care more about the enthusiasm of the owner, which is the emotional experience.

**Fig 21:**
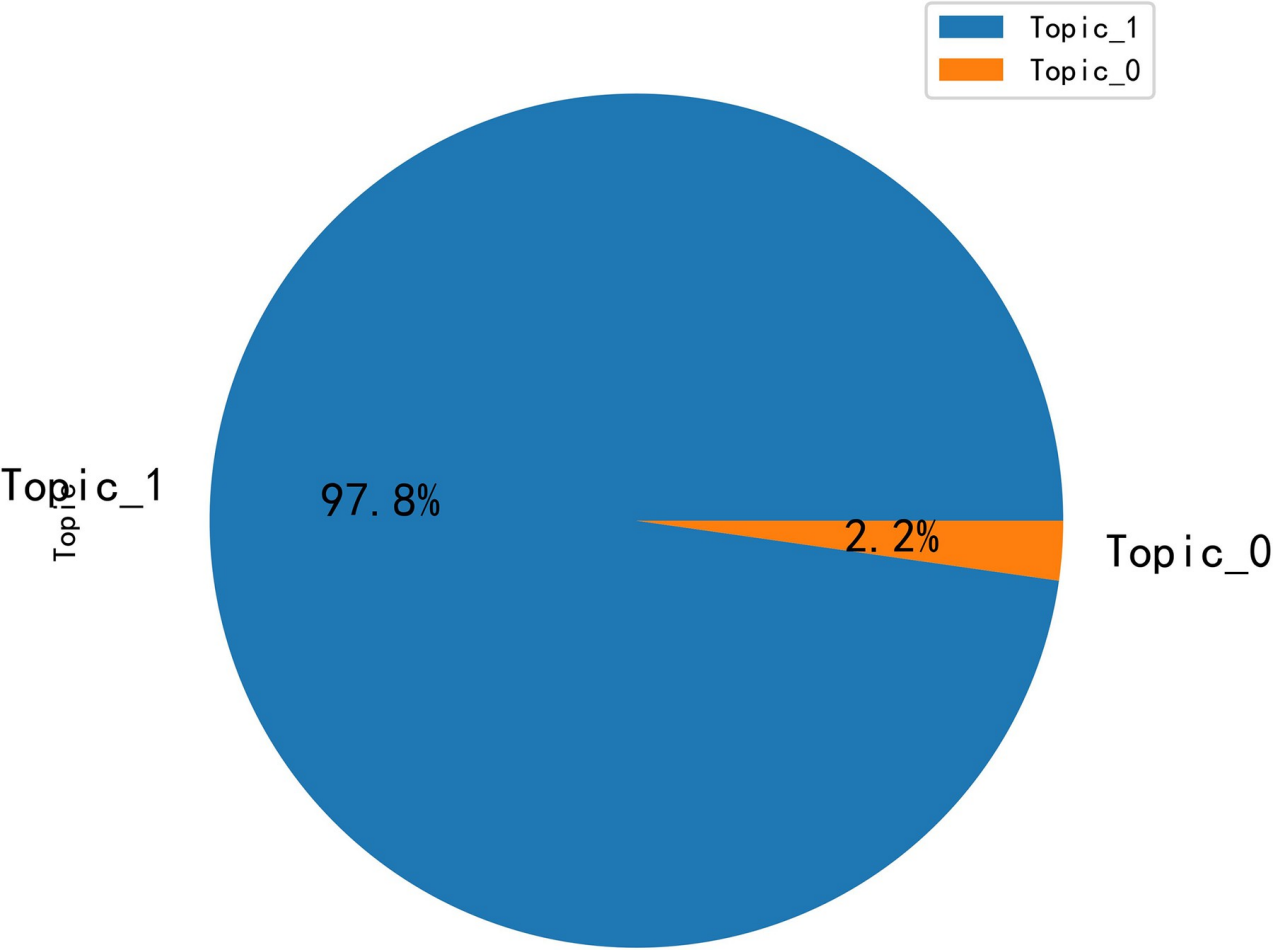
Negative sentiment review topic intensity distribution graph.

The results of the analysis of Bert’s topic words for the negative emotional evaluation are shown in Table 5, and the topic intensity map is shown in Fig 21.

**Table 5:** “Topic-word” distribution and weighting of negative sentiment reviews.

topic 0	weights	topic 1	weights
Average	1.815444003	Rooms	0.077365929
Bad	0.757936829	Hotel	0.058230754
No	0.448938275	Owner	0.053729278
Late	0.448938275	Service	0.038601581
Asking questions	0.448938275	Enthusiasm	0.037620373
Privacy	0.448938275	Guest House	0.036278233
Help	0.448938275	B&B	0.034148186
Poor goods	0.448938275	Evening	0.031114593
Bad	0.448938275	Environment	0.031114593
Half an hour	0.448938275	Breakfast	0.028789063

According to the topic-intensity distribution chart, topic 1 accounts for 97.8%, which provides an important reference for optimizing B&B management. According to the topic-word distribution map, it is concluded that topic 1 is the emotional characteristics of B&B, and boss, service, and enthusiasm are among the keywords. Combining the data as negative emotional data, word cloud diagram, and semantic network diagram, it can be concluded that the main cause of the negative emotion of tourists is the poor emotional experience, i.e., the lack of enthusiasm and service; and the importance of these causes greatly exceeds the non-essential objective conditions of B&B.

### Summary

As a result, this paper constructs a framework for B&B review analysis based on Bert model and machine learning methods such as CNN+BiLSTM and CNN+BiGRU, in which Bert model performs topic analysis, CNN+BiLSTM and CNN+BiGRU models are used for sentiment analysis, and topic extraction provides content for optimal management and sentiment analysis provides direction for it. The models provide optimization ideas and rectification directions for the management of Yunnan B&B in the context of rural revitalization. In the context of rural revitalization, tourism income is an important part of residents’ income generation and the government’s economic development, and B&Bs play a significant role in it, so the management optimization of B&Bs is a top priority. First, as the first destination for tourists, the first impression of B&B is very important, not only will it affect the evaluation of tourists for the B&B, but also affect the evaluation of tourists for the local tourism place, and then affect the tourist experience and consumption desire of tourists, and then affect the B&B operators, tourism product operators, residents, and local economy, so the management optimization of B&B should control the first impression well. Secondly, tourists do not have high requirements for the basic facilities of B&Bs, cleanliness, neatness, and hygiene are the most important parts for tourists, which are also the most basic requirements for hotels, and tourists’ requirements for Yunnan B&Bs are to meet the basic requirements (cleanliness and hygiene). This is not a reasonable direction to optimize the management of B&Bs. What tourists care most about is a warm attitude, attentive service, and a friendly B&B operator (owner). A friendly, warm attitude will make people happy, the attitude of the B&B operator is the most intuitive feeling of tourists about the local customs, and the help provided by the B&B operator will largely improve the tourist experience. The results of the data analysis point to one word, "boss," indicating that the focus of the management optimization of B&Bs at this stage is "people management": the government should strengthen the management of B&B operators to promote quality operation; excellent B&B operators should also focus on improving The government should strengthen the management of B&B operators to promote the quality operation, and good B&B operators should also make efforts to improve their service attitude, put themselves in the shoes of tourists, and strive to enhance the emotional experience of tourists. Thirdly, B&Bs that are in a position to do so can differentiate themselves with the help of local special resources or their resources to make their B&Bs more attractive to tourists, but they must ensure that tourists’ requirements for the objective conditions of B&Bs (cleanliness and hygiene) and tourists’ requirements for B&B operators (enthusiasm and good service).

## An evolutionary game theory model of rural B&B development

This paper uses machine learning algorithms to provide an accurate analysis of the role of B&Bs in the development of rural tourism. It determines that the main stakeholders are the government, B&B operators, and tourists, and highlights the critical factor influencing the tourist experience as the management of B&Bs, with a focus on service and a warm attitude.

### Definition of core stakeholders

Rural tourism has become an important source of economic growth in underdeveloped areas, including local villages in Yunnan, and is now highly valued by government management agencies at all levels. As a crucial component of the core stakeholders in rural tourism, the government can influence the decision-making process of other stakeholders through various means, such as policy-making, planning, incentives, and image building, and ultimately influence the direction of rural tourism development. Government management agencies have considerable political capital advantages and are therefore considered the key drivers of rural tourism development.

Residents of rural communities involved in tourism business activities are directly responsible for providing services to tourists, and the quality of their services and their attitudes are key elements that shape tourists’ experiences. Residents are considered important stakeholders in the tourism industry [34], as they play an important role in preserving the natural and cultural resources of rural tourism destinations. Involving local residents in tourism activities can promote tourism development, and their attitudes toward tourism can inform planning and the development of appropriate tourism products [59]. Encouraging friendly business practices among local residents can also improve tourists’ perceptions of tourism.

Through the analysis of tourist behavior in rural tourism destinations, it has been found that tourists are the primary contributors to rural tourism [60]. Tourists are the most important consumer group in the rural tourism market and are key stakeholders in rural tourism development. They are interested in the rural tourism experience, making them the primary focus of the industry. They are an essential element of the logistics, information flow, and human flow processes in rural tourism and are the main subjects of rural tourism activities. The quality of tourists’ rural tourism experience has a significant impact on whether rural tourism can achieve sustainable development. As consumers, they play an essential role in the development of the industry by promoting positive word-of-mouth, increased awareness, and revenue.

Tourist satisfaction is critical to the growth of the industry, and their preferences for rural tourism experiences and service quality should be addressed. The transfer of power from the seller to the buyer has revolutionized the world economy, and the importance of the consumer in the development of the industry cannot be overemphasized. Government intervention is needed to guide the provision of tourism services that meet the needs of tourists and thereby enhance their experience [61]. Tourists are critical stakeholders who seek to achieve good tourism perceptions and benefit claims during the tourism process [32]. The tourism development process must consider the needs of tourists and satisfy them through competitive destination marketing programs that meet their aspirations [62]. The tourism industry provides tourists with cognitive, social, and emotional benefits and allows them to relax and gain knowledge [63, 64]. The tourist experience is also essential for destination branding [65].

Government agencies at all levels are primarily interested in several aspects of rural tourism development. These include enhancing the visibility of rural tourism destinations and creating destination brands, increasing local financial revenues, promoting local economic growth, addressing employment issues for local residents, and improving their living standards. In addition, establishing a fair benefit-sharing mechanism to ensure that all stakeholders can benefit equitably, protecting local material, natural, and intangible cultural heritage resources, achieving local sustainable development, and improving the overall economic, environmental, and cultural aspects to achieve rural revitalization are important priorities for government management agencies.

B&B operators play a crucial role in the development of rural tourism as they directly benefit from and contribute to it. However, their business activities have a significant impact on the environment and economy of rural tourism destinations. They are considered to be the business subjects of rural tourism and need the support of government agencies at all levels to operate successfully. In return, they are expected to operate with integrity and provide quality tourism products and services to tourists. B&B operators are important stakeholders in rural tourism and have the right to operate and manage tourism businesses. They are key players in driving economic growth and generating revenue for the government through taxes. Their demands include earning high profits, receiving supportive policies and training opportunities, having the right to operate independently and participate in tourism decision-making, establishing fair rules for revenue distribution, maintaining good relations with all stakeholders, increasing the visibility of rural tourism destinations and attracting more tourists.

Tourists are at the center of rural tourism development, and their demands guide the direction of the industry as a whole. Government, tour operators, and local communities all have a role to play in shaping tourists’ perceptions and experiences of rural tourism destinations. A successful tourism process should provide quality experiences for visitors, who in turn can become advocates for rural tourism and help promote positive images of these destinations. If rural tourism products lack uniqueness and quality, visitors may have a negative experience and leave negative reviews. In the age of social media, the impact of negative word-of-mouth can be particularly damaging, underscoring the importance of meeting tourists’ needs and expectations. Visitors are looking for high-quality, authentic experiences that highlight local culture and heritage while ensuring sustainable development and conservation of natural resources. They also want value for money, personal and property security, and the freedom to choose their activities without feeling pressured to buy.

### Conflict of interest between parties

There is a significant conflict of interest among B&B operators, as the problem of homogenization is widespread. In their quest for economic gain, tourism operators prioritize projects that can quickly generate profits, resulting in the simultaneous implementation of several similar or identical projects in adjacent areas. This trend leads to a proliferation of low-quality duplicate structures that diminish the perceived value of tourism to visitors.

One issue that arises between governments at all levels and B&B operators is a conflict of interest. B&B operators tend to prioritize their economic gain when making decisions about B&B development, often prioritizing immediate profits over long-term benefits. This short-term profit cycle can threaten the sustainability of rural tourism sites. Governments, on the other hand, prioritize the public interest, seeking to improve overall well-being, increase economic income, and achieve sustainable development. To prevent B&B operators from cutting corners or deceiving tourists, government agencies need to strictly enforce regulations and carry out supervisory functions to ensure the quality of rural tourism products and services.

The conflicts of interest between tourists and B&B operators can be categorized into two types. First, there is a conflict of economic interests, as tourists seek affordable and quality tourism services, while B&B operators aim to maximize profits at minimal costs. Second, there is a conflict of objectives, as tourists want a quality tourism experience, but too many tourists can negatively affect their experience. In contrast, B&B operators prioritize high numbers of tourists to increase their profits.

The successful development of rural tourism depends on the cooperation and collaboration of three main stakeholders: the government, B&B operators, and tourists. However, conflicts of interest can arise within and between these groups. Each stakeholder has its own goals and interests, which may not always coincide. Therefore, all stakeholders must work together, actively participate, and engage in effective communication and cooperation to achieve mutual benefits. Through evolutionary game theory, each stakeholder can adjust their strategies and find optimal solutions to resolve conflicts of interest and promote the healthy development of rural tourism.

### Basic assumptions and related parameters

As core stakeholders choose their strategies, they will continuously adjust their strategies according to the strategies of other subjects of interest, which is a dynamic game process that is continuously optimized over time. To simplify the model, the following assumptions are made:

According to the previous analysis, this study identifies the main stakeholders involved in the evolutionary game as managers, tourism service providers, and tourists. Specifically, management, B&B operators (owners), and tourists (visitors who stay in B&Bs).

In this study, the core stakeholders of rural tourism, including the government, B&B operators, and tourists, are assumed to exhibit "limited rationality" in the evolutionary game process. This means that each stakeholder follows a traditional behavioral strategy and analyzes the existing strategies of other stakeholders. They rely on inertia in choosing their strategies, do not predict the future, and are influenced by exogenous factors. In addition, each party has incomplete information and will not find the best strategy at the beginning. Throughout the game, players go through a complex and long process of learning, imitation, trial, and error to adjust their strategies and gradually determine the best approach.

Tourists are primarily concerned with obtaining a quality tourism experience. They can adopt two strategies for the development of rural B&Bs. The first strategy is to support the overall development of the B&B industry, denoted as T_11_, while the second strategy is not to support the development of rural B&Bs, denoted as T_12_. Tourist support for rural B&B development is probabilistic, where the probability of supporting rural tourism development is represented by P_1_, and the probability of not supporting rural tourism development is represented by 1 – P_1_ (where P_1_ is a value between 0 and 1). Thus, the set of strategies available to tourists for rural B&B development is {T_11_, T_12_}.

For tourists to support the development of rural B&Bs, they need to provide honest and high-quality reviews on B&B review websites and help B&B operators and management to develop and optimize the industry. By supporting the development of rural B&Bs, tourists gain emotional benefits and recognition, denoted as R_1_, but they also incur time costs, denoted as C_1_. When B&B operators choose quality management strategies, tourists receive emotional rewards, such as seeing their favorite B&Bs improve and building friendships with the operators, denoted as R_12_. In addition, they may receive rewards from the government or platforms when management intervenes, denoted as R_13_.

In the scenario where tourists decide not to support the development strategy of the rural B&B industry, there are no benefits or costs associated with their decision. However, if B&B operators do not implement a quality business strategy, they will incur losses from poor tourist experiences, which is recorded as L_12_.

B&B operators play a critical role in rural tourism development and are highly invested in pursuing better economic benefits, while also considering other factors. B&B operators have two strategies to choose from when developing their business: the first strategy is quality operation, denoted as B_11_, with probability of P_2_; the second strategy is non-quality operation, denoted as B_12_, with probability of 1-P_2_ (P_2_∈[0,1]). The set of strategies available to B&B operators for rural B&B development is {B_11_, B_12_}.

According to the considerations of the B&B operators, the choice of a quality management strategy entails an additional cost C_21_ in addition to the necessary operating costs C_2_ but also results in a benefit R_2_. If the management chooses a management strategy, the B&B operator will receive a benefit R_23_, and if tourists support the development of a quality rural B&B industry, it will receive a benefit R_21_. On the other hand, if the B&B operator chooses a non-quality management strategy, it pays only the basic operating costs C_2_ and gains R_2_. However, the B&B operator suffers a loss L_23_ if the management chooses a management strategy, and a loss L_21_ if tourists support the development of the B&B industry, which could potentially lead to a reduction in tourists.

The government, as a manager, plays a crucial role in the development of rural tourism, to achieve precise poverty alleviation and rapid economic growth in rural areas. The provision of basic public services and the implementation of various policies and management regulations are among how the government realizes resource allocation and carries out public governance. In areas where the overall economic situation is already in a good state of development, the government may not provide financial support for the development of rural tourism, and there may be no corresponding policy orientation or preferential conditions for attracting investment. For the development of rural tourism, the administration has two strategies for managing B&Bs. The first strategy is management, which is designated as G_11_, while the second strategy is non-management, which is designated as G_12_. To maximize benefits, the government weighs the benefits and risks of each strategy and chooses between them with a certain probability. The probability that the government chooses to intervene in the development of the rural B&B industry is P_3_, while the probability that it chooses not to intervene is 1-P_3_, where P_3_ is in the range [0,1]. The government’s strategy for rural B&B development is {G_11_, G_12_}.

Based on Management Considerations. When management chooses a management strategy, it must pay management costs C_3_ and receive benefits that improve the local economy, etc. R_3_. If B&B operators choose to operate with quality, they must pay similar costs such as rewards R_23_; if B&B operators choose non-quality strategies, they receive corresponding fines, etc., recorded as L_23_. If tourists choose to support rural B&B development, the management has to pay additional costs R_13_, while receiving positive help from tourists for local economic and social development R_31_. If the management chooses non-management, the management has no costs to pay but has to face the development loss caused by the disruption of the B&B industry, recorded as L_3_.

### Evolutionary game model design and analysis of stable strategies

#### Evolutionary game model designing

According to the assumptions above, we can get the utility matrices of government, B&B operators and tourists, shown in Table 6.

**Table 6:** Utility matrix of the tripartite game model of government, B&B operators, and tourists.

Strategy Set	Tourists	B&B operators	Government
(*T*_11_, *B*_11_, *G*_11_)	*R*_1_−*C*_1_+*R*_13_+R_12_	*R*_2_−*C*_2_−*C*_21_+*R*_23_+*R*_21_	*R*_3_−*C*_3_−*R*_23_−*R*_13_+*R*_31_
(*T*_11_, *B*_11_, *G*_12_)	*R*_1_−*C*_1_+*R*_12_	*R*_2_−*C*_2_−*C*_21_+*R*_21_	−*L*_3_
(*T*_11_, *B*_12_, *G*_11_)	*R*_1_−*C*_1_+*R*_13_	*R*_2_−*C*_2_−*L*_23_−*L*_21_	*R*_3_−*C*_3_+*L*_23_−*R*_13_+*R*_31_
(*T*_11_, *B*_12_, *G*_12_)	*R*_1_−*C*_1_	*R*_2_−*C*_2_−*L*_21_	−*L*_3_
(*T*_12_, *B*_11_, *G*_11_)	0	*R*_2_−*C*_2_−*C*_21_+*R*_23_	*R*_3_−*C*_3_−*R*_23_
(*T*_12_, *B*_11_, *G*_12_)	0	*R*_2_−*C*_2_−*C*_21_	−*L*_3_
(*T*_11_, *B*_12_, *G*_11_)	−*L*_12_	*R*_2_−*C*_2_−*L*_23_	*R*_3_−*C*_3_+*L*_23_
(*T*_12_, *B*_12_, *G*_12_)	−*L*_12_	*R*_2_−*C*_2_	−*L*_3_

#### Analysis of replication dynamic equations and stable strategies of tourists

Let the expected benefit of tourists supporting the development of rural B&B be *U*_11_ and the expected benefit of non-supporting the development of rural B&B be *U*_12_.


$$U_{11}=P_{2}P_{3}(R_{1}-C_{1}+R_{13}+R_{12})+P_{2}(1-P_{3})(R_{1}-C_{1}+R_{12})+(1-P_{2})P_{3}(R_{1}-C_{1}+R_{13})+(1-P_{2})(1-P_{3})(R_{1}-C_{1})=P_{2}R_{12}+P_{3}R_{13}+R_{1}-C_{1}$$ (8)

$$U_{12}=(1-P_{2})P_{3}(-L_{12})+(1-P_{2})(1-P_{3})(-L_{2})=P_{2}L_{12}-L_{12}$$ (9)

Replicator dynamic equation is,

$$F(P_{1})=\frac{dP_{1}}{dt}=P_{1}(1-P_{1})(P_{2}R_{12}+P_{3}R_{13}+R_{1}-C_{1}-P_{2}L_{12}+L_{12})$$ (10)

When $P_{2}=\frac{-P_{3}R_{13}-R_{1}+C_{1}-L_{12}}{R_{12}-L_{12}},$ F(P_1_) ≡ 0, which means that all states are stable states.

When $P_{2}\neq \frac{-P_{3}R_{13}-R_{1}+C_{1}-L_{12}}{R_{12}-L_{12}}$, let F(P_1_) = 0, which gives P1 = 0 and P1 = 1 as two steady states. The derivative of F(P_1_) gives $F^{\prime }(P_{1})=(1-2P_{1})(P_{2}R_{12}+P_{3}R_{13}+R_{1}-C_{1}-P_{2}L_{12}+L_{12})$.

At this point there are two cases:

When $P_{2}>\frac{-P_{3}R_{13}-R_{1}+C_{1}-L_{12}}{R_{12}-L_{12}},$ P1 = 1 is the equilibrium point, and tourists choose to participate in supporting the rural B&B improvement strategy as an evolutionary stable strategy.

When $P_{2}<\frac{-P_{3}R_{13}-R_{1}+C_{1}-L_{12}}{R_{12}-L_{12}},$ P1 = 0 is the equilibrium point, and tourists choose not to support the rural B&B improvement strategy is an evolutionary stable strategy.

**Fig 22:**
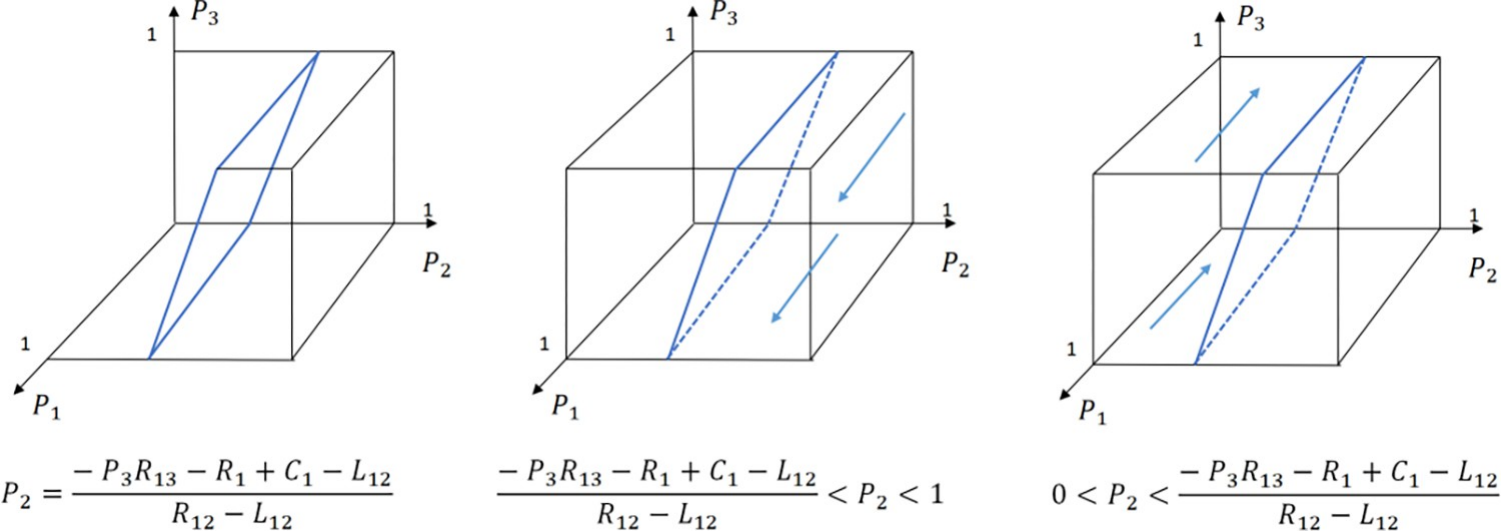
Evolutionary process of tourists in different situations.

The evolution of the tourists’ decision is shown in Fig 22.

#### Analysis of replication dynamic equations and stable strategies of B&B operator

Let the expected benefit of a B&B operator choosing a quality business strategy be U_21_ and the expected benefit of choosing a non-quality business strategy to be U_22_.


$$U_{21}=P_{1}P_{3}(R_{2}-C_{2}-C_{21}+R_{23}+R_{21})+P_{1}(1-P_{3})(R_{2}-C_{2}-C_{21}+R_{21})+(1-P_{1})P_{3}(R_{2}-C_{2}-C_{21}+R_{23})+(1-P_{1})(1-P_{3})(R_{2}-C_{2}-C_{2}1)=P_{1}R_{21}+P_{3}R_{23}+R_{2}-C_{2}-C_{21})$$ (11)

$$U_{22}=P_{1}P_{3}(R_{2}-C_{2}-L_{23}-L_{21})+P_{1}(1-P_{3})(R_{2}-C_{2}-L_{21})+(1-P_{1})P_{3}(R_{2}-C_{2}-L_{23})+(1-P_{1})(1-P_{3})(R_{2}-C_{2})=-P_{1}L_{21}-P_{3}L_{23}+R_{2}-C_{2}$$ (12)

Replicator dynamic equation is,

$$F(P_{2})=\frac{dP_{2}}{dt}=P_{2}(1-P_{2})(P_{1}R_{21}+P_{3}R_{23}-C_{21}+P_{1}L_{21}+P_{3}L_{23})$$ (13)

When $P_{3}=\frac{-(R_{21}+L_{21})P_{1}+C_{21}}{R_{23}+L_{23}},$ F(P_2_) ≡ 0, which means that all states are stable states.

When $P_{3}\neq \frac{-(R_{21}+L_{21})P_{1}+C_{21}}{R_{23}+L_{23}}$, let F(P_2_) = 0, which gives P2 = 0 and P2 = 1 as two steady states. The derivative of F(P_2_) gives $F^{\prime }(P_{2})=(1-2P_{2})(P_{1}R_{21}+P_{3}R_{23}-C_{21}+P_{1}L_{21}+P_{3}L_{23})$.

At this point there are two cases:

When $P_{3}>\frac{-(R_{21}+L_{21})P_{1}+C_{21}}{R_{23}+L_{23}},$ P_2_ = 1 is the equilibrium point, and the B&B operator chooses a quality business strategy as the evolutionary stabilization strategy.

When $P_{3}<\frac{-(R_{21}+L_{21})P_{1}+C_{21}}{R_{23}+L_{23}},$ P_2_ = 0 is the equilibrium point, and the B&B operator chooses the non-quality business strategy as the evolutionary stabilization strategy.

**Fig 23:**
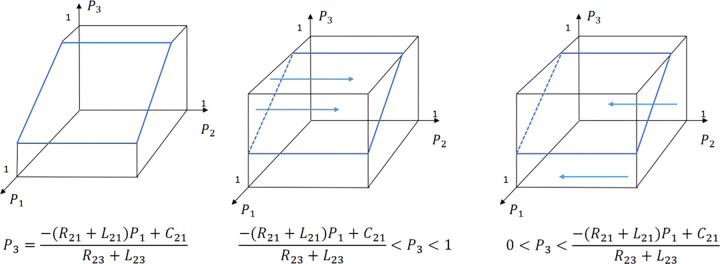
Evolutionary process of B&B operator in different situations.

The evolution of the B&B operator’ decision is shown in Fig 23.

#### Analysis of replication dynamic equations and stable strategies of government

Let the expected benefit of the government choosing to manage the B&B industry be U_31_ and the expected benefit of choosing non-management be U_32_.


$$U_{31}=P_{1}P_{2}(R_{3}-C_{3}-R_{23}-R_{13}+R_{31})+P_{1}(1-P_{2})(R_{3}-C_{3}+L_{23}-R_{13}+R_{31})+(1-P_{1})P_{2}(R_{3}-C_{3}-R_{23})+(1-P_{1})(1-P_{2})(R_{3}-C_{3}+L_{23})=-P_{1}R_{13}+P_{1}R_{31}-P_{2}R_{23}+R_{3}-C_{3}+L_{23}-P_{2}L_{23}$$ (14)

$$U_{32}=-P_{1}P_{2}L_{3}+P_{1}(1-P_{2})(-L_{3})+(1-P_{1})P_{2}(-L_{3})+(1-P_{1})(1-P_{2})(-L_{3})=-L_{3}$$ (15)

Replicator dynamic equation is,

$$F(P_{3})=\frac{dP_{3}}{dt}=P_{3}(1-P_{3})(P_{1}R_{31}-P_{1}R_{13}-P_{2}R_{23}+R_{3}-C_{3}+L_{23}-P_{2}L_{23}+L_{3})$$ (16)

When $P_{2}=\frac{P_{1}R_{31}-P_{1}R_{13}+R_{3}-C_{3}+L_{23}+L_{3}}{R_{23}+L_{23}},$ F(P_3_) ≡ 0, which means that all states are stable states.

When $P_{2}\neq \frac{P_{1}R_{31}-P_{1}R_{13}+R_{3}-C_{3}+L_{23}+L_{3}}{R_{23}+L_{23}}$, let F(P_3_) = 0, which gives P_3_ = 0 and P_3_ = 1 as two steady states. The derivative of F(P_3_) gives $F^{\prime }(P_{1})=(1-2P_{3})(P_{1}R_{31}-P_{1}R_{13}-P_{2}R_{23}+R_{3}-C_{3}+L_{23}-P_{2}L_{23}+L_{3})$.

At this point there are two cases:

When $P_{2}<\frac{P_{1}R_{31}-P_{1}R_{13}+R_{3}-C_{3}+L_{23}+L_{3}}{R_{23}+L_{23}},$ P_3_ = 1 is the equilibrium point, and the government chooses management of the B&B industry strategy as the evolutionary stable strategy.

When $P_{2}>\frac{P_{1}R_{31}-P_{1}R_{13}+R_{3}-C_{3}+L_{23}+L_{3}}{R_{23}+L_{23}},$ P_3_ = 0 is the equilibrium point, and the government chooses non-management of the B&B industry strategy as the evolutionary stable strategy.

**Fig 24:**
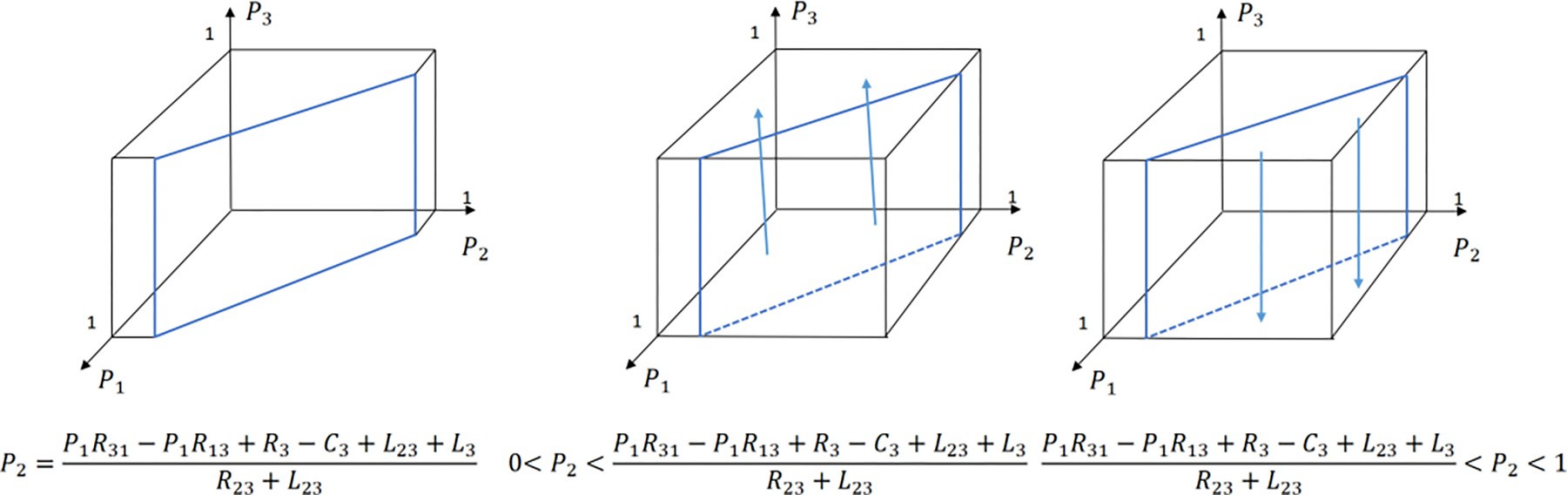
Evolutionary process of government in different situations.

The evolution of the government’ decision is shown in Fig 24.

#### Analysis of the evolutionary stability of each participant’s strategy

According to Lyapunov stability theory, the system at the equilibrium point the asymptotic stability of the system can be determined by analyzing the characteristics of the Jacobian matrix of the system values to determine, and the Jacobian matrix is obtained for this system as follows.


$$J=\left|\begin{array}{ccc} \frac{dF(P_{1})}{dP_{1}} & \frac{dF(P_{1})}{dP_{2}} & \frac{dF(P_{1})}{dP_{3}}\\ \frac{dF(P_{2})}{dP_{1}} & \frac{dF(P_{2})}{dP_{2}} & \frac{dF(P_{2})}{dP_{3}}\\ \frac{dF(P_{3})}{dP_{1}} & \frac{dF(P_{3})}{dP_{2}} & \frac{dF(P_{3})}{dP_{3}} \end{array}\right|=\left|\begin{array}{ccc} a & P_{1}(1-P_{1})(R_{12}-L_{12}) & P_{1}(1-P_{1})R_{13}\\ P_{2}(1-P_{2})(R_{21}+L_{21}) & b & P_{2}(1-P_{2})(R_{23}+L_{23})\\ P_{3}(1-P_{3})(R_{31}-R_{13}) & P_{3}(1-P_{3})(-R_{23}-L_{23}) & c \end{array}\right|$$

$$where\;a=(1-2P_{1})(P_{2}R_{12}+P_{3}R_{13}+R_{1}-C_{1}-P_{2}L_{12}+L_{12})$$

$$b=(1-2P_{2})(P_{1}R_{21}+P_{3}R_{23}-C_{21}+P_{1}L_{21}+P_{3}L_{23})$$

$$c=(1-2P_{3})(P_{1}R_{31}-P_{1}R_{13}-P_{2}R_{23}+R_{3}-C_{3}+L_{23}-P_{2}L_{23}+L_{3})$$

Given that the asymptotically stable solution of a dynamical system of a multi-group evolutionary game must be a strict Nash equilibrium solution, in addition to E_1_ (0,0,0), E_2_ (0,0,1), E_3_ (0,1,0), E_4_ (1,0,0), E_5_ (0,1,1), E_6_ (1,0,1), E_7_ (1,1,0), and E_8_ (1,1,1), the rest are not in an asymptotically stable state. Substitute the above 8 points into the Jacobi matrix and solve for the matrix eigenvalues.

The matrix eigenvalues are solved and the results are shown in Table 7.

**Table 7:** Eigenvalues of Jacobian matrix.

Equilibrium points	Eigenvalue *λ*_1_	Eigenvalue *λ*_2_	Eigenvalue *λ*_3_
*E*_1_(0,0,0)	*R*_1_−*C*_1_+*L*12	−*C*_21_	*R*_3_−*C*_3_+*L*_23_+*L*_3_
*E*_2_(0,0,1)	*R*_13_+*R*_1_−*C*_1_+*L*12	*R*_23_−*C*_21_+*L*_23_	−*R*_3_+*C*_3_−*L*_23_−*L*_3_
*E*_3_(0,1,0)	*R*_12_+*R*_1_−*C*_1_	*C*_21_	−*R*_23_+*R*_3_−*C*_3_+*L*_3_
*E*_4_(1,0,0)	−*R*_1_+*C*_1_−*L*_12_	*R*_21_−*C*_21_+*L*_21_	*R*_31_−*R*_13_+*R*_3_−*C*_3_+*L*_3_+*L*_23_
*E*_5_(0,1,1)	*R*_12_+*R*_13_+*R*_1_−*C*_1_	−*R*_23_+*C*_21_+*L*_23_	*R*_23_−*R*_3_+*C*_3_−*L*_3_
*E*_6_(1,0,1)	−*R*_13_−*R*_1_+*C*_1_−*L*_12_	*R*_21_+*R*_23_−*C*_21_+*L*_21_+*L*_23_	−*R*_31_−*R*_13_−*R*_3_+*C*_3_−*L*_3_−*L*_23_
*E*_7_(1,1,0)	−*R*_12_−*R*_1_+*C*_1_	−*R*_21_−*R*_23_+*C*_21_−*L*_21_−*L*_23_	*R*_31_−*R*_13_−*R*_23_+*R*_3_−*C*_3_+*L*_3_
*E*_8_(1,1,1)	−*R*_12_−*R*_13_+*C*_1_−*R*_1_	−*R*_21_−*R*_23_+*C*_21_−*L*_21_−*L*_23_	−*R*_31_+*R*_13_+*R*_23_−*R*_3_+*C*_3_−*L*_3_

To simplify the analysis of the eigenvalue signs for different equilibrium points, we assume that the benefits of cooperation between the government, B&B operators, and tourists to promote quality rural tourism development are greater than the benefits of not cooperating to improve rural tourism management. $R_{1}-C_{1}+R_{13}+R_{12}>-L_{12},\;R_{2}-C_{2}-C_{21}+R_{23}+R_{21}>R_{2}-C_{2},\;R_{3}-C_{3}-R_{23}-R_{13}+R_{31}>-L_{3},\;R_{1}$ and C_1_ should be almost equal. The eigenvalues obtained for each equilibrium point are shown in Table 8, where the values in parentheses represent the sign of the eigenvalues, and "*" indicates that the sign cannot be determined. According to the Lyapunov method, if an equilibrium point is asymptotically stable, all eigenvalues of its corresponding Jacobian matrix must be negative. Therefore, the stability of each equilibrium point can be determined from its eigenvalues.

**Table 8:** Stability analysis of equilibrium points.

Equilibrium points	Eigenvalue *λ*_1_	Eigenvalue *λ*_2_	Eigenvalue *λ*_2_	Stability
*E*_1_(0,0,0)	+	-	*	Unstable point
*E*_2_(0,0,1)	+	+	*	Unstable point
*E*_3_(0,1,0)	+	+	*	Unstable point
*E*_4_(1,0,0)	-	+	+	Unstable point
*E*_5_(0,1,1)	+	+	*	Unstable point
*E*_6_(1,0,1)	-	*	*	Unknown
*E*_7_(1,1,0)	-	*	+	Unstable point
*E*_8_(1,1,1)	-	-	-	ESS

The table above shows that the equilibrium point E_6_(1,0,1) is an evolutionary stable point when R_21_+R_23_−C_21_+L_21_+L_23_<0 and −R_31_−R_13_−R_3_+C_3_−L_23_−L_3_>0. At this point, tourists support the improvement of the rural B&B industry and government regulations, but B&B operators do not choose the quality operation strategy. This is because the cost of choosing the quality operation strategy is much higher than the benefits received from the government and tourists when operating in a quality way. In addition, the loss of potential tourists and the fines imposed by the government for operational problems further outweigh the benefits. However, at the equilibrium point E_8_(1,1,1), the benefits of supporting the development of the rural B&B industry are substantial for all stakeholders, including the government, B&B operators, and tourists, who are committed to optimizing the management of rural B&Bs, developing the industry, and improving the quality of rural tourism.

## Numerical simulation based on system dynamics

In this part, to further verify the validity of the evolutionary game model and to clarify the relationship between various influencing factors and the stability of the system, we simulated the dynamic evolutionary process. The evolutionary trajectory was simulated and analyzed using MATLAB software.

### Simulation of the evolution of a tripartite subject game system

In 2022, rural tourism in the province welcomed an impressive total of 300 million tourists, generating a substantial tourism revenue of 188.872 billion yuan [66]. As a result, the average expenditure per tourist exceeded 600 yuan. This highlights the potential impact of substandard business operations, referred to as *L*_21_, which resulted in a loss of 6 from potential tourists. Without improvements in the tourism landscape, it is postulated that tourists would experience further negative travel outcomes, both financial and emotional. This dimension of negative travel experiences is encapsulated in *L*_12_, which is set at 7. Consequently, tourist support for B&B optimization efforts can be cultivated by taking into account aspects such as travel costs, overall well-being and quality of travel experiences, resulting in the assignment of *R*_1_ with a value of 10. In addition, the benefits to government go beyond tourism expenditure and include tax revenue, credibility and other facets. Consequently, *R*_3_ is assigned a value of 10 to reflect the comprehensive benefits that government derives from the tourism sector. According to official documents from the Yunnan government, the province is currently providing subsidy funds to support the development of rural B&Bs. Consequently, the financial commitment of the government is slightly higher than the expected benefits, leading to the assignment of C_3_ with a value of 12 [67]. To encourage greater involvement of tourists in optimizing the management of B&Bs in Yunnan, while at the same time providing incentives to tourists, *R*_12_ is set with a value of 11 [68]. This measure aims to encourage the active participation of tourists. In addition, it is important to recognize that B&Bs that run quality operations have to incur significant expenses, including essential hardware upgrades, resulting in significant cost outlays. In line with practical considerations, these costs are estimated to be around twice the value of *C*_3_. To be consistent with the findings of the previous analysis, *C*_21_ is set at 20 or 30.

It is important to emphasize that the simulated system evolves within a virtual time frame, denoted "t", as opposed to real-time conditions. In addition, certain parameters have been rounded to facilitate numerical simulations. The configuration of other parameters for different scenarios is detailed in Table 9, based on real-world considerations, input from rural B&B practitioners, and consultation with relevant experts.

**Table 9:** The setting of exogenous variables (BAU means business as usual).

Exogenous variables	BAU1	BAU2	Exogenous variables	BAU1	BAU2
*R*_12_	11	-	*L*_23_	7	-
*R*_13_	4	-	*L*_3_	6	-
*R*_1_	10	-	*R*_21_	5	-
*C*_1_	18	-	*R*_23_	7	-
*L*_12_	7	-	*C*_21_	30	20
*L*_21_	6	-	*R*_31_	8	-
*R*_3_	10	-	*C*_3_	12	-

The exogenous variables are set as seen in Table 9, which includes four baseline scenarios (i.e. BAU1 and BAU2) corresponding to the two ESS in last section. The change of parameters is to verify the stability of the strategies. ‘–’ means that the parameters do not change compared with BAU1.

**Fig 25:**
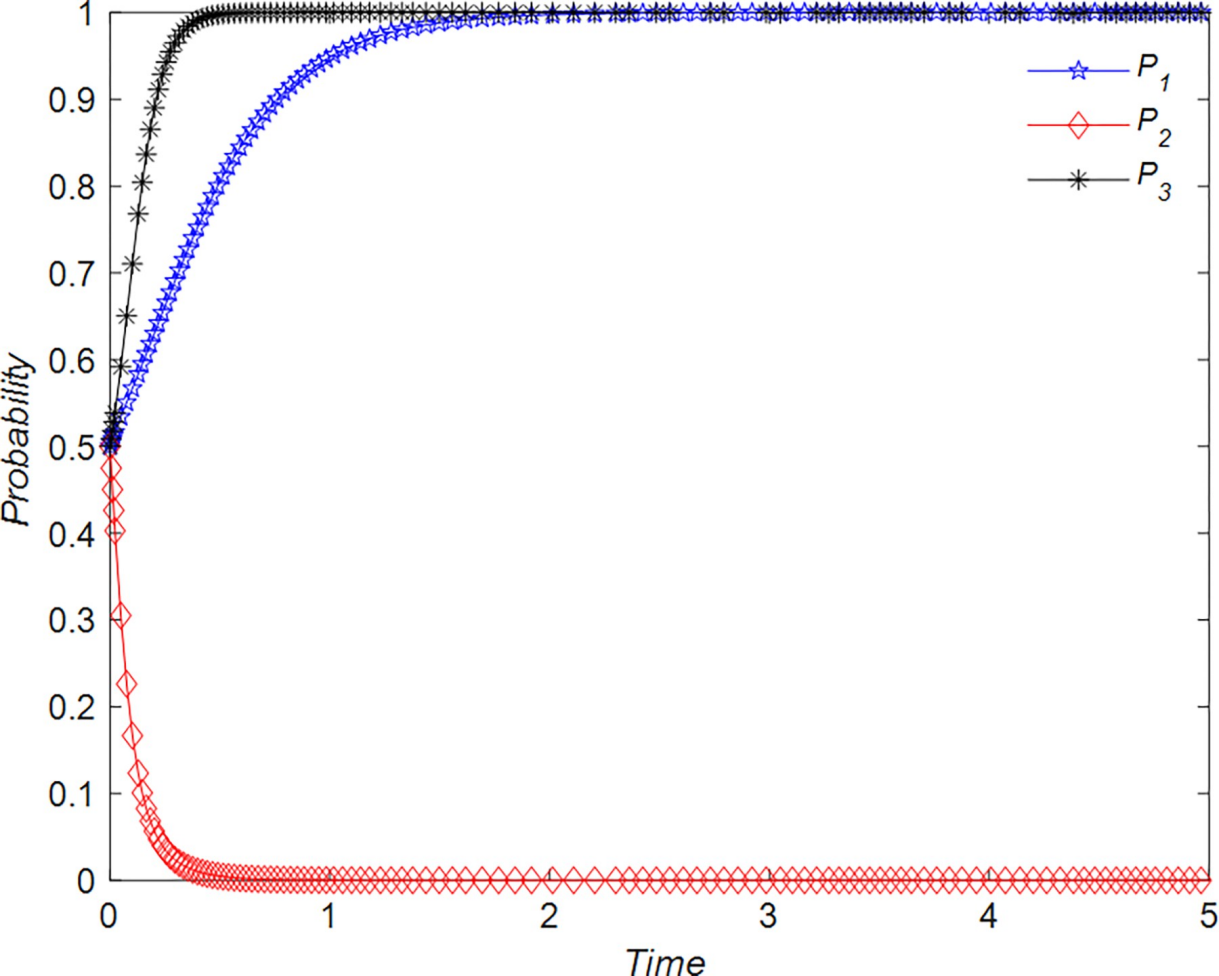
Stability analysis when ESS is (1, 0, 1).

The strategy (1, 0, 1) corresponds to the parameters of the BAU1 scenario. The dynamic evolution process is depicted in Fig 25, which shows that tourists, as consumers, are inclined to support the development of the rural B&B industry to enhance their travel experience. In response, the government is also motivated to regulate the B&B industry as an important component of the local economy to promote rural revitalization. However, the government’s management capacity is limited and its investment in the development of the B&B industry is insufficient. As a result, there are no incentives for tourists to support the B&B industry, nor are there specific measures to help B&B operators improve their service quality. In addition, the government does not impose strict penalties on B&B operators for problematic behavior, such as overcharging, due to a lack of effective management. As a result, the additional cost for B&B operators to adopt quality operation strategies is too high, which leads them to choose non-quality operation strategies. In addition, it takes longer for tourists to reach a stable state, indicating the inefficiency of the government as a whole.

**Fig 26:**
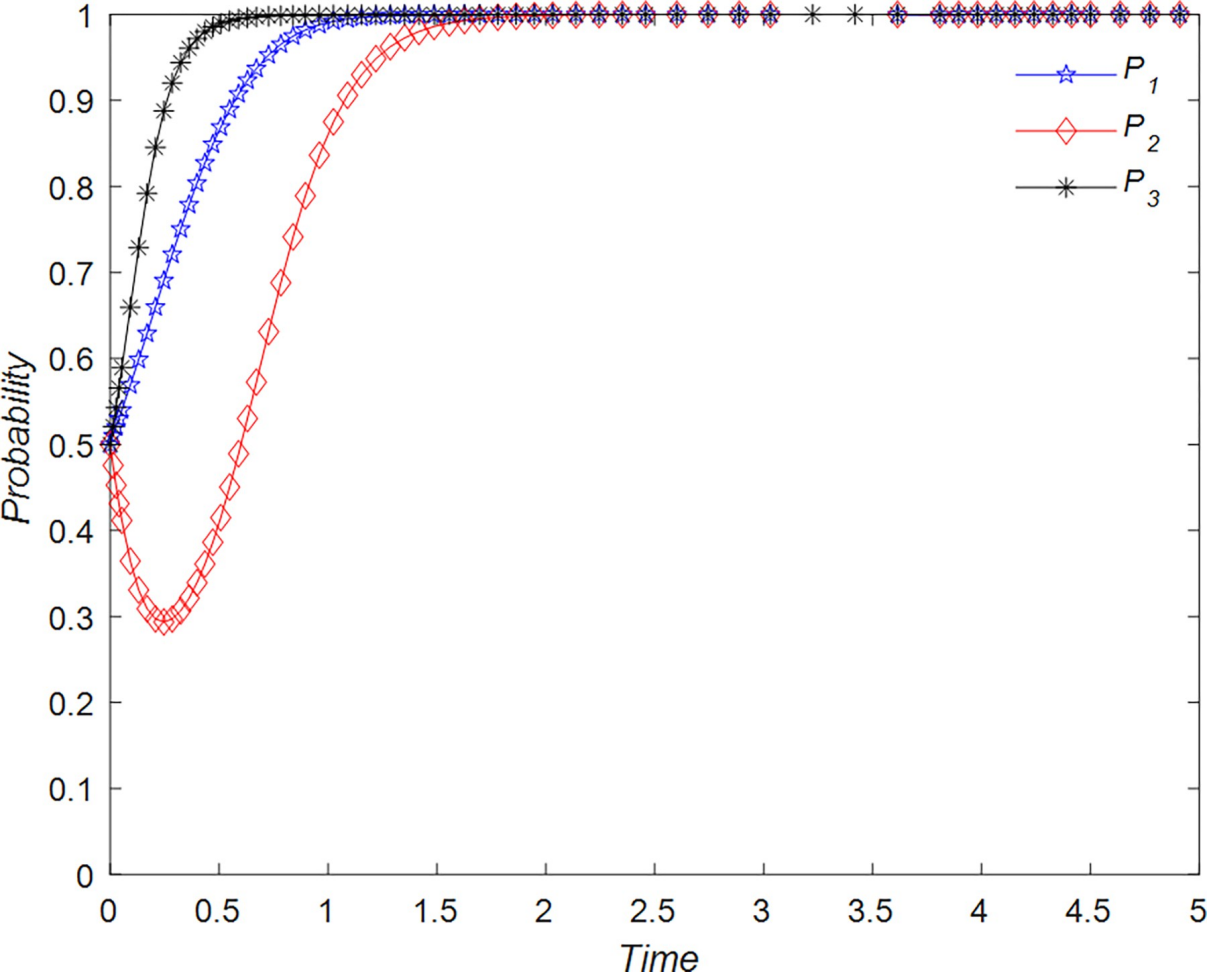
Stability analysis when ESS is (1, 1, 1).

The strategy (1, 1, 1) corresponds to the parameters under BAU2. The dynamic evolution process is shown in Fig 26. The study shows that tourists, B&B operators, and the government can achieve a synergistic ideal situation of cooperation to optimize B&B management and improve the quality of rural revitalization. Many strategic influences cause all three parties to choose to positively influence rural tourism development, mainly depending on the government’s management efforts, the government’s investment in the development of the rural B&B industry, and the income of B&B operators when they choose to operate with quality. Both the government and tourists decided to support the development of the rural B&B industry for a certain period. The B&B management started to choose the strategy of non-quality operation and then chose the strategy of quality operation. On the one hand, B&B operators needed a certain period to observe the market situation to determine that optimizing B&B management was a beneficial strategy for them. On the other hand, B&B operators also need to spend some time and effort to implement quality management. After that, the three parties reach a steady state. In addition, the efficiency of the government as an overall organization is reflected in the fact that it reaches a steady state more quickly.

### The influence of exogenous variables

In this section, several analyses are performed to evaluate the influence of different exogenous variables on the participants of the game model. These analyses are based on the BAU2 scenario, which represents the evolutionary stability point of the system at (1, 1, 1).

#### The influence of additional costs in the case of quality operation of B&B operators

**Fig 27:**
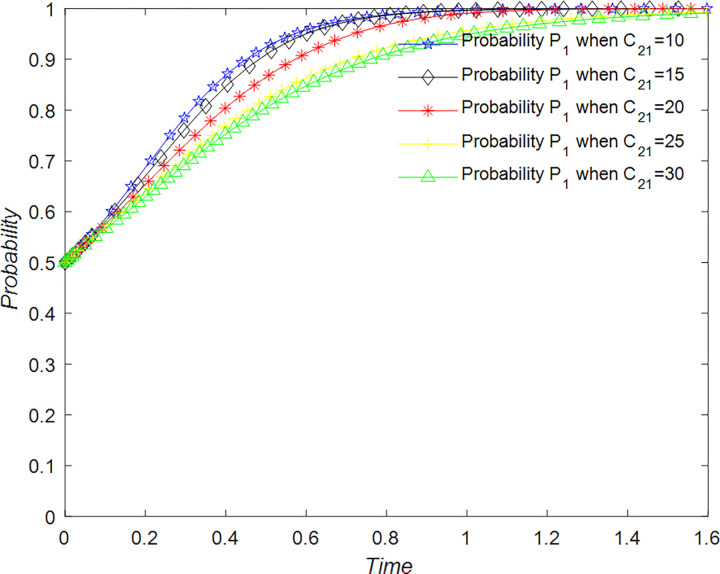
The influence of C_21_ on P_1_.

**Fig 28:**
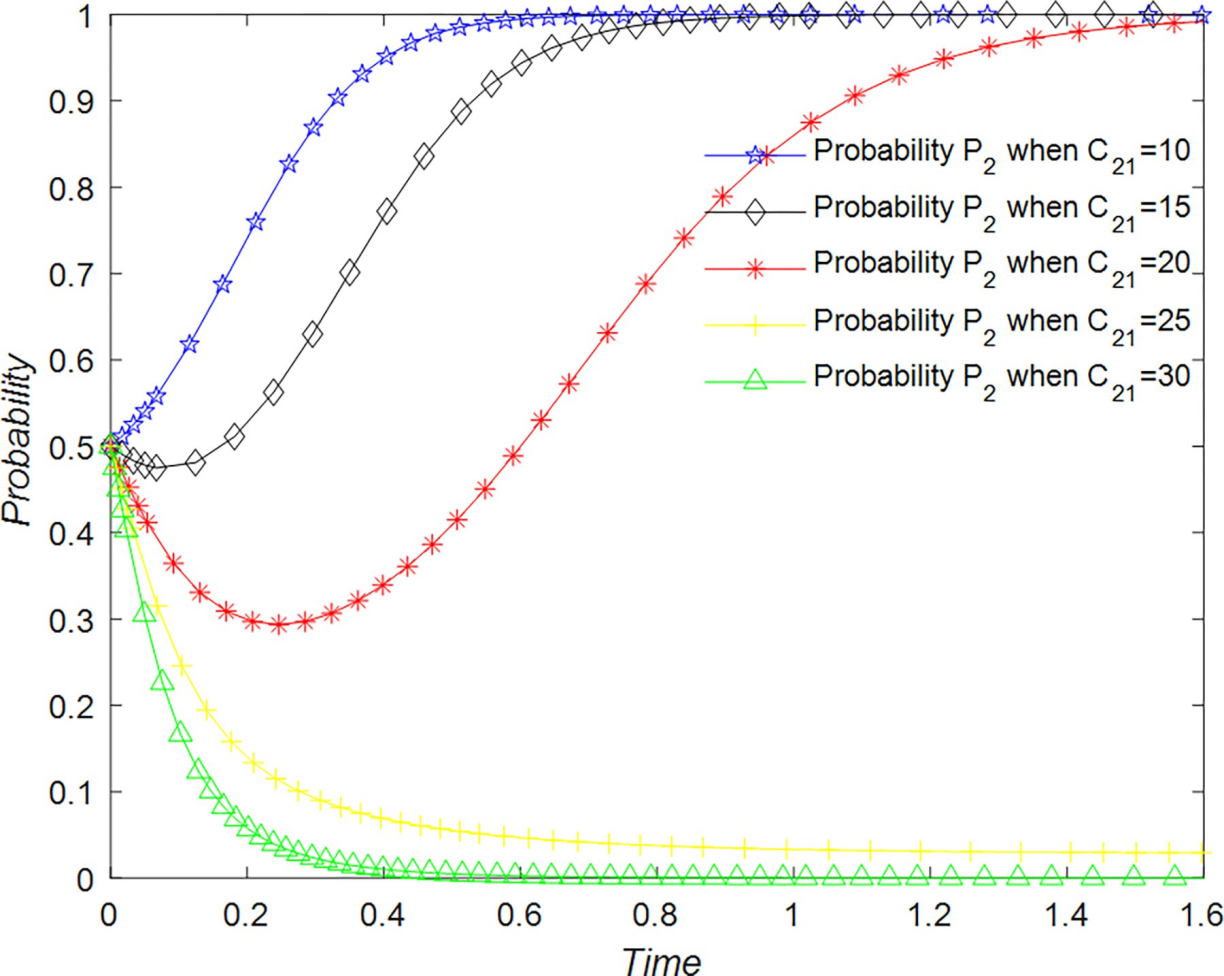
The influence of C_21_ on P_2_.

**Fig 29:**
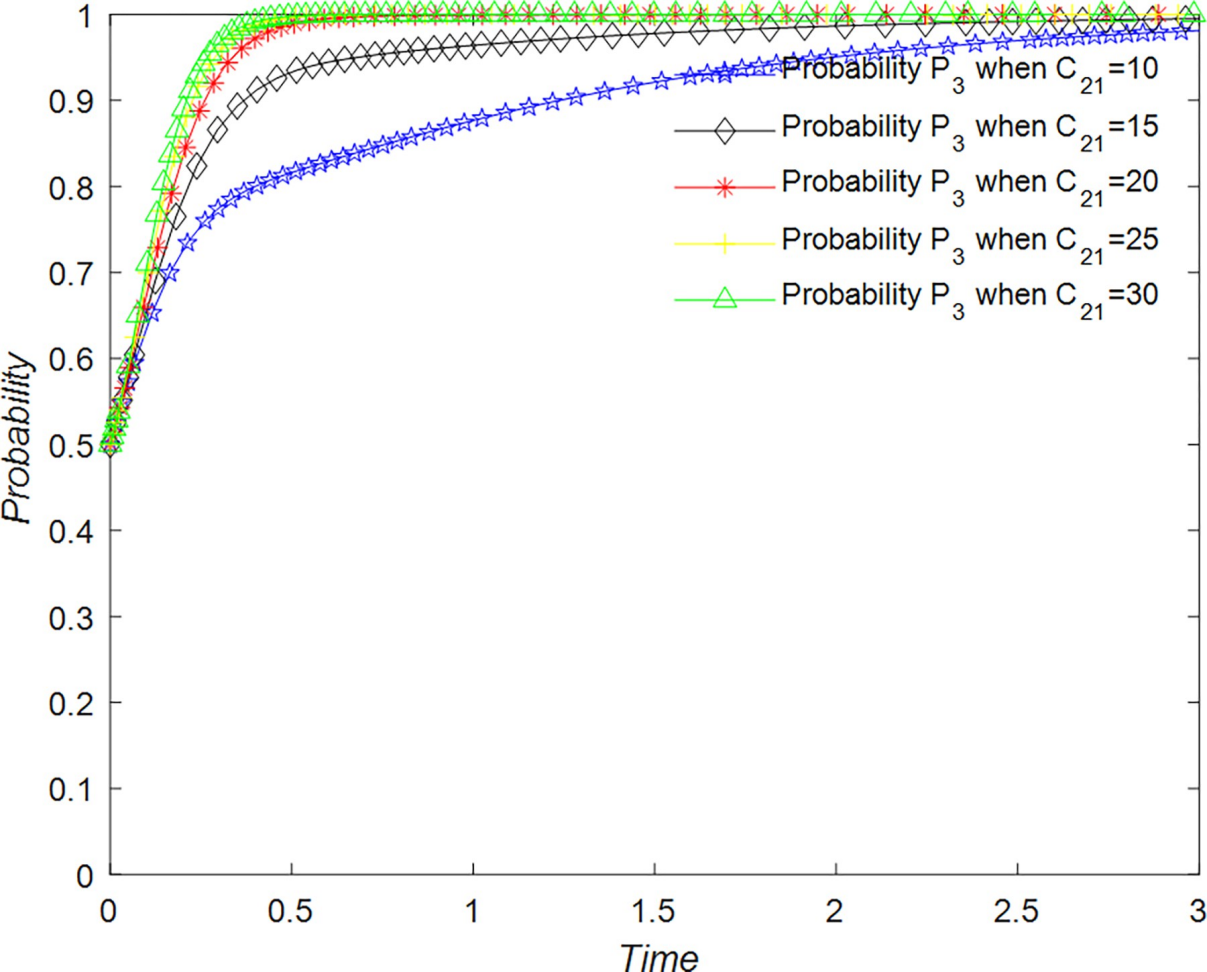
The influence of C_21_ on P_3_ Influence of losses to the local economy when the government does not manage (L_3_).

The decision to run a B&B with a focus on quality involves various additional costs such as time, money, and effort. In this study, the influence of different levels of C_21_, which represents the additional costs incurred by B&B operators, on the decision of the three parties was analyzed through simulations using values of 10, 15, 20, 25, and 30. Figs 27–29 show the effects of these additional costs on P_1_, P_2_, and P_3_, respectively. The results showed that tourists consistently supported the optimal management of B&Bs regardless of the variation in C_21_. However, larger values of C_21_ resulted in a slower evolution towards a steady state on the tourist side, as the high additional costs paid by B&B operators may already provide a satisfactory experience for tourists, leading to a lower level of attention paid to local B&B management. Similarly, the government may ultimately choose to manage the B&B industry and reach a steady state. In particular, when C_21_ is particularly small, the government evolves more slowly toward a steady state because B&B operators are more likely to operate with quality due to the negligible additional cost, leading to less urgency for the government to intervene in B&B management.

The decision of B&B operators to adopt a quality operations strategy is significantly influenced by the extra costs involved, including time, money and human costs. In scenarios where C_21_ is low, B&B operators are more likely to adopt quality operations due to the low costs and potential profits, contributing to rural revitalization. As C_21_ increases, B&B operators may initially shift to non-quality operations and later transition to quality operations based on market conditions and cost-benefit analysis. The higher the C_21_, the longer it takes for B&B operators to transition to quality operations, and when C_21_ is particularly high, B&B operators may choose to operate only with non-quality operations. The paper highlights that reasonable additional operating costs can help optimize B&B management and promote rural revitalization.

**Fig 30:**
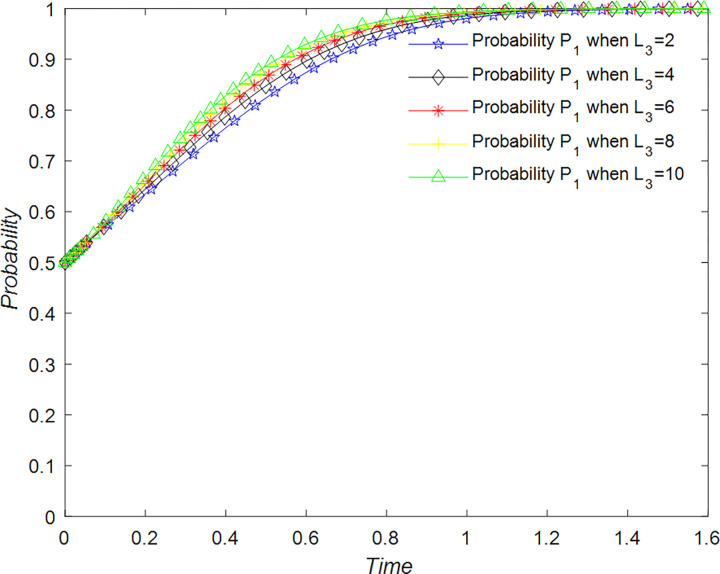
The influence of L_3_ on P_1_.

**Fig 31:**
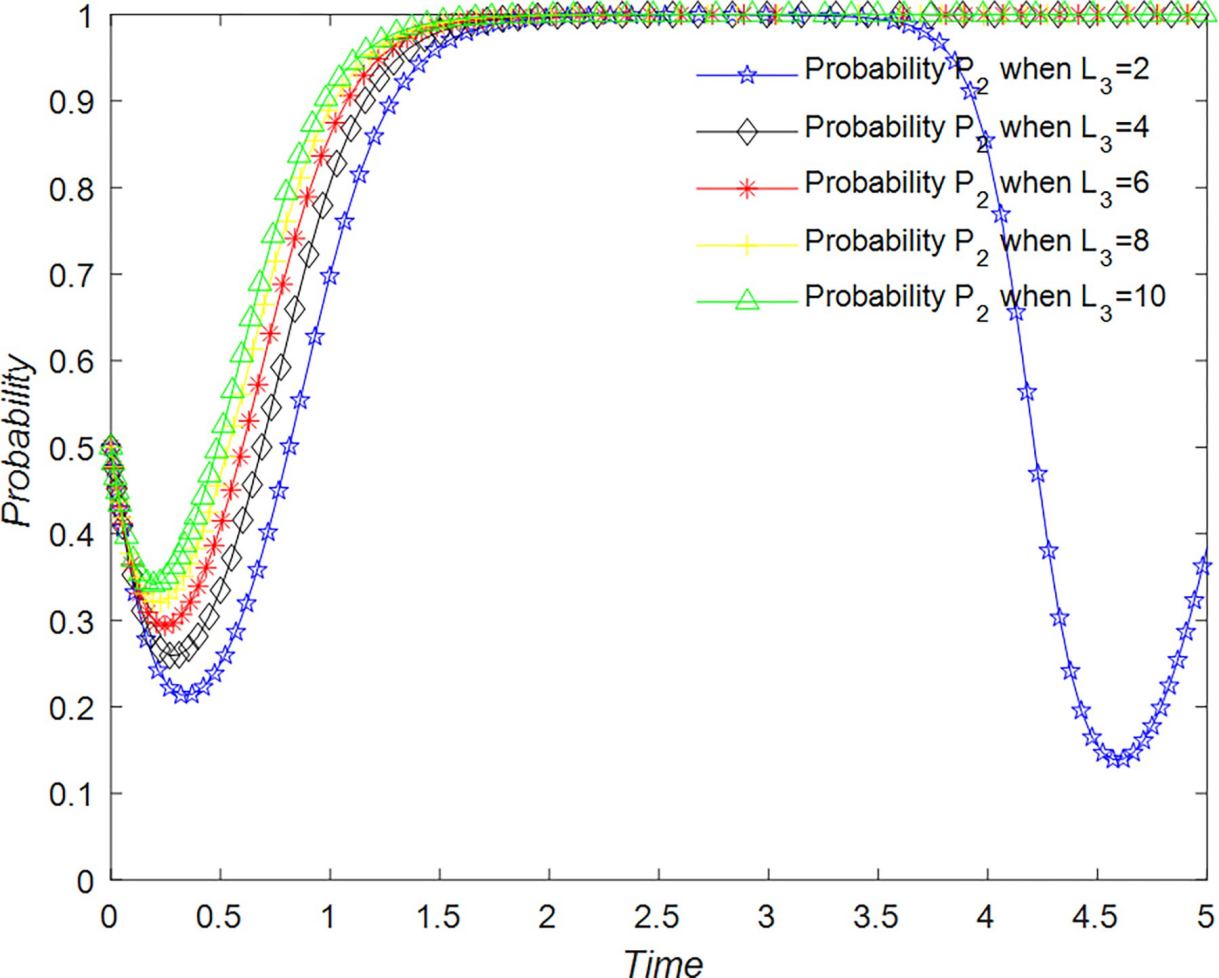
The influence of L_3_ on P_2_.

**Fig 32:**
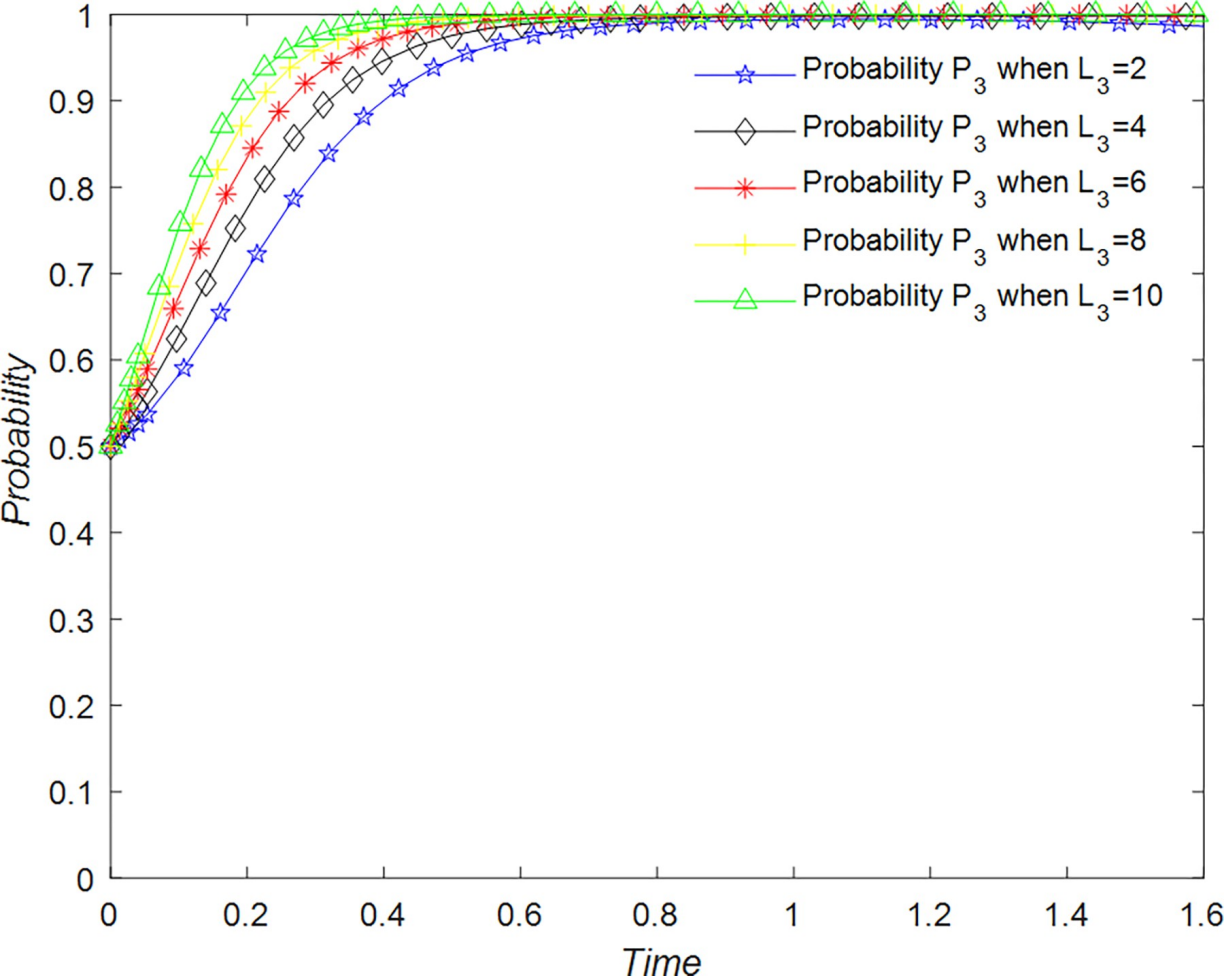
The influence of L_3_ on P_3_.

The local B&B industry has a significant impact on local economic income and rural revitalization in Yunnan’s rural areas. Therefore, the government aims to manage the local B&B industry and support local operators to optimize its management. To explore the influence of local economic loss on stakeholders in this game model, this paper conducts simulation analysis using different values of L_3_, namely 2, 4, 6, 8, and 10. Figs 30–32 illustrate the influence of local economic loss on P_1_, P_2_, and P_3_, respectively.

The results of the study indicate that the variation in L_3_, which represents the economic loss suffered by the local community due to the absence of B&B management, does not have a significant impact on tourists’ decision-making. This is because tourists do not consider local economic income as a deciding factor when choosing a B&B. However, for the government, the magnitude of the economic loss associated with a no-management strategy directly affects its intervention in the B&B industry. In particular, the greater the economic loss to the local community, the more urgent the government’s involvement in B&B management becomes since promoting local economic development is a top priority for the government.

B&B operators tend to choose non-premium business strategies initially because of the delayed impact of declining economic income. This is because they tend to choose less costly business practices in the beginning. However, the impact of the economic decline eventually causes them to change, and the larger the economic loss, the faster they are likely to change strategies. If the economic loss is relatively small, it may not be significant enough to influence the B&B operator’s strategy choice, resulting in oscillation between the two strategies. Therefore, a larger L_3_ may facilitate tripartite cooperation.

#### The influence of the loss of the next tour experience, which is also bad when tourists do not support the management optimization of rural B&Bs (L_12_)

**Fig 33:**
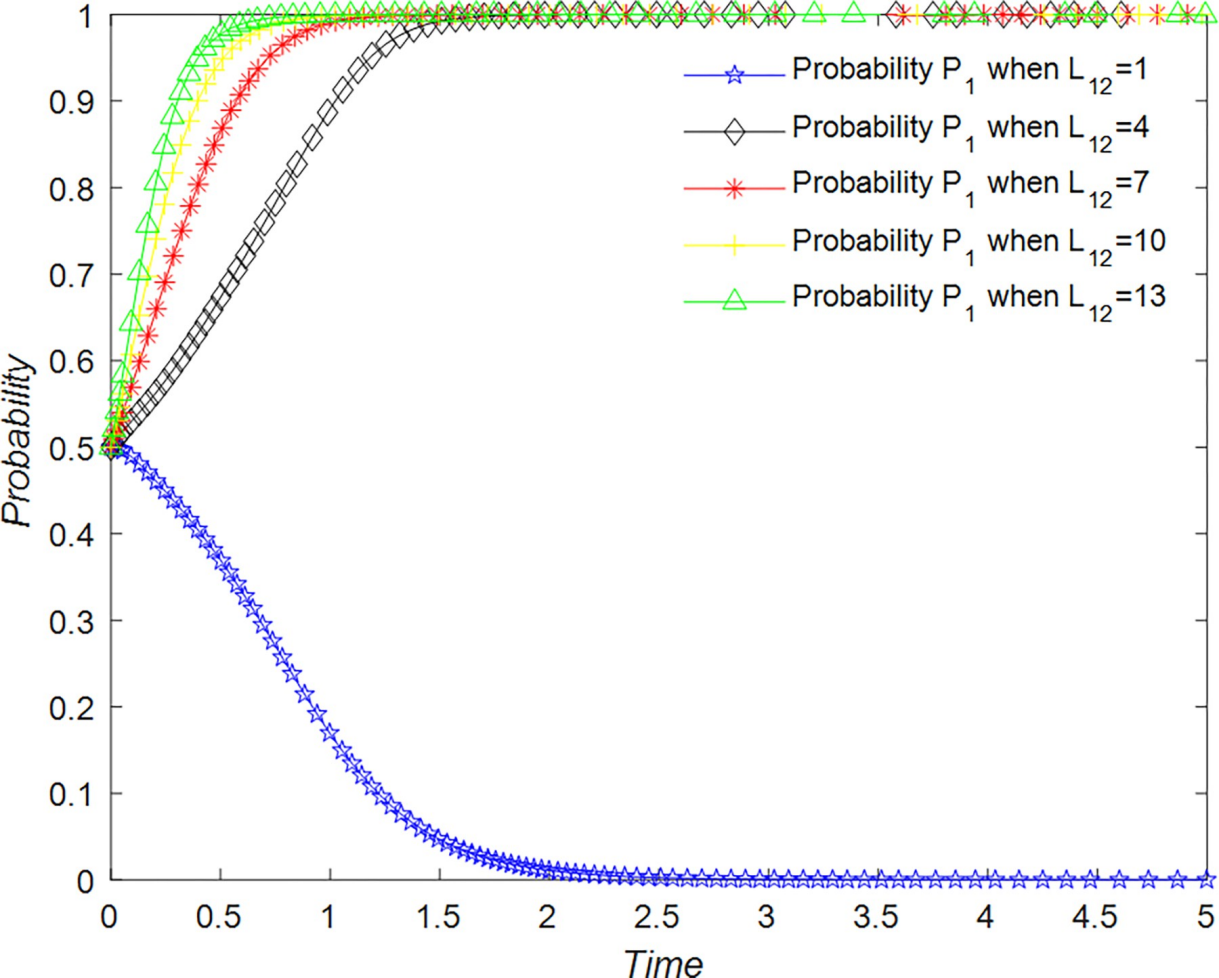
The influence of L_12_ on P_1_.

**Fig 34:**
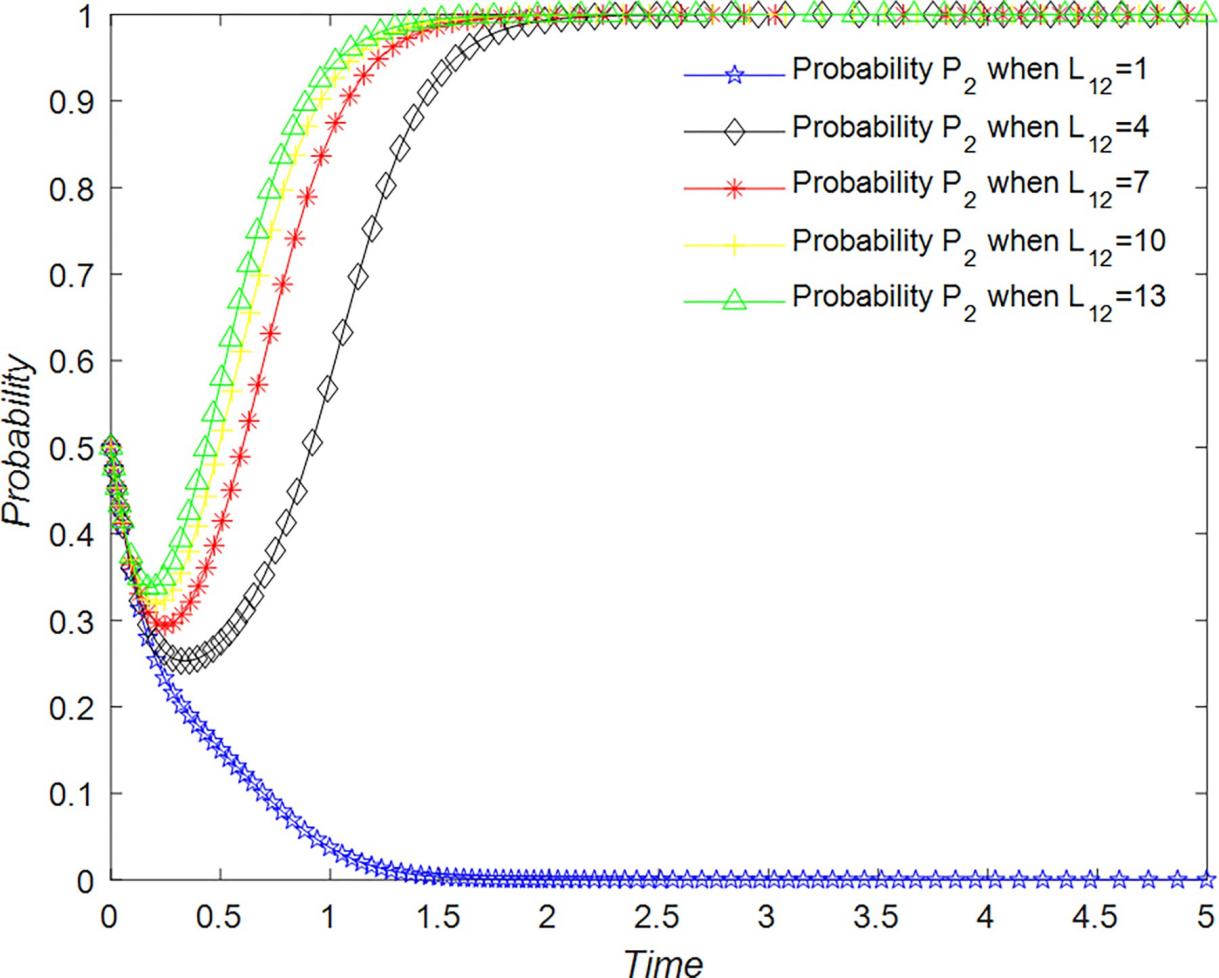
The influence of L_12_ on P_2_.

**Fig 35:**
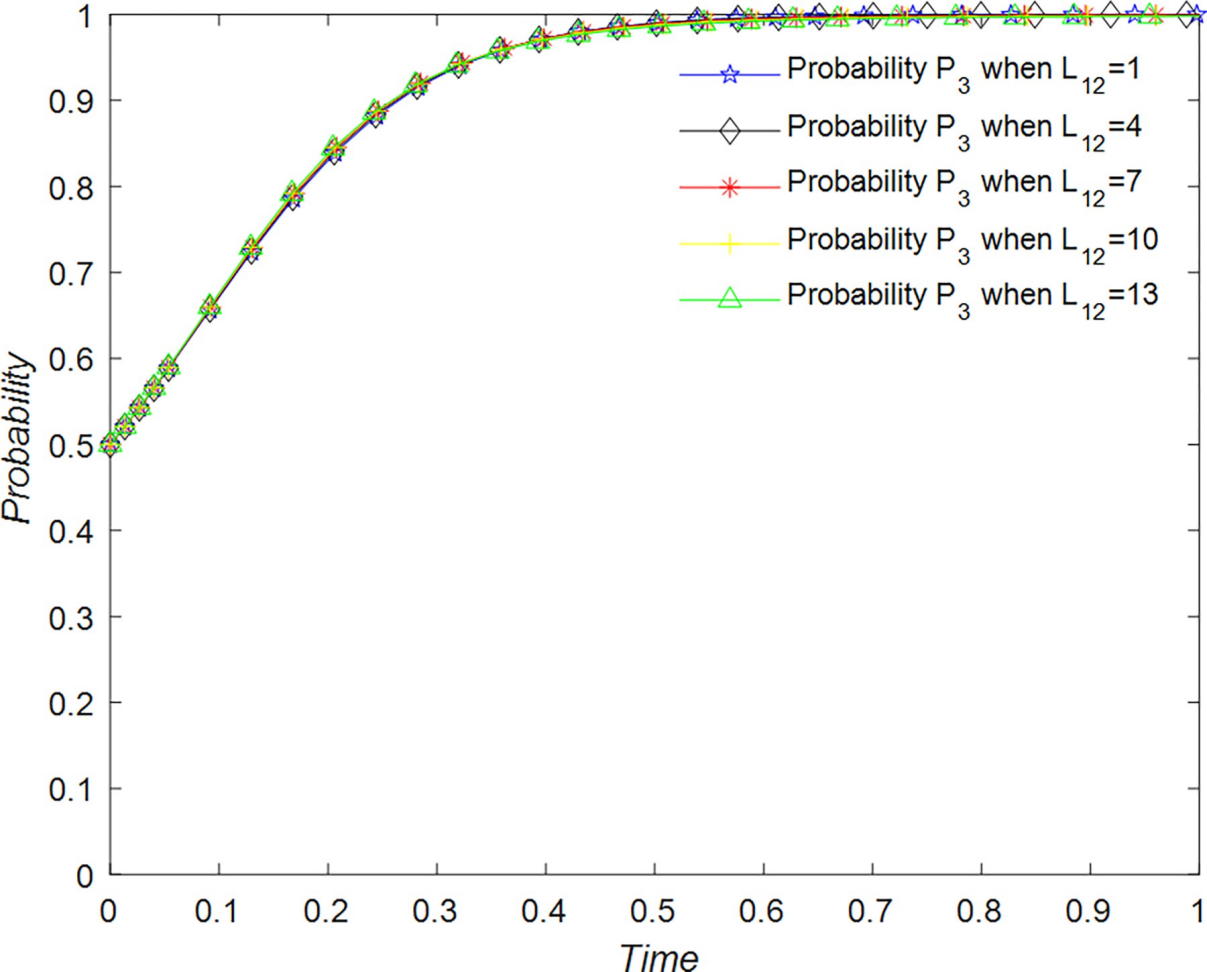
The influence of L_12_ on P_3_.

In cases where tourists are not in favor of improving the management of rural B&Bs, and both B&B operators and the government do not take steps to address this issue, tourists are likely to experience a negative tourism environment, which may lead to a negative experience in their subsequent trips. To explore this scenario, this paper conducts a simulation analysis with L_12_ values of 1, 4, 7, 10, and 13. Figs 33–35 illustrate the influence of L_12_ on P_1_, P_2_, and P_3_, respectively.

The larger the L_12_ value, the greater the likelihood that tourists will support improvements in rural B&B management, while the smaller the L_12_ value, the better the next trip experience, resulting in a lack of motivation for tourists to support B&B improvements. B&B operators initially chose a non-quality management strategy to minimize costs but eventually shifted to a quality strategy to achieve evolutionary stability. This is due to the negative impact of a poor next-trip experience on B&B revenues. In cases where L_12_ is particularly small, i.e. does not affect the tourist’s multiple trips, B&B operators prefer to adopt a non-quality management strategy. The government must manage the local B&B industry and promote rural revitalization. The loss of L_12_ for tourists does not influence the government’s decision. Therefore, a higher L_12_ value is beneficial for the development of the rural B&B industry.

#### Influence of loss of visitors (L_21_) caused by government regulation to B&Bs when they choose non-quality operations

**Fig 36:**
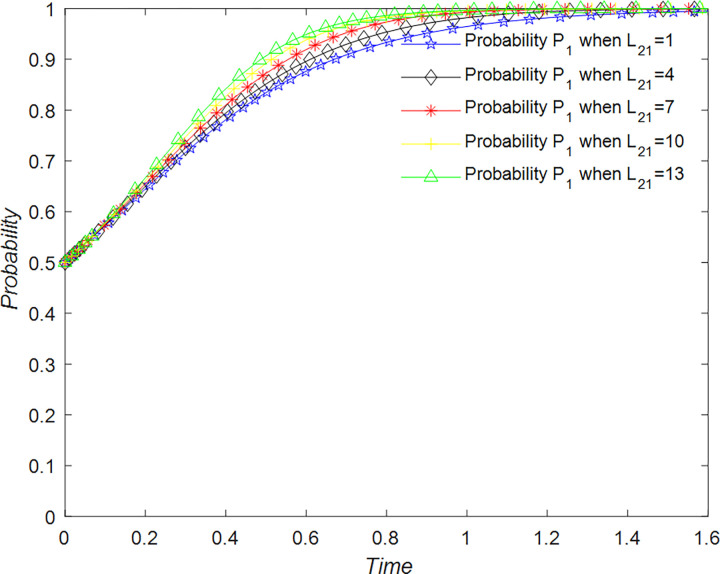
The influence of L_21_ on P_1_.

**Fig 37:**
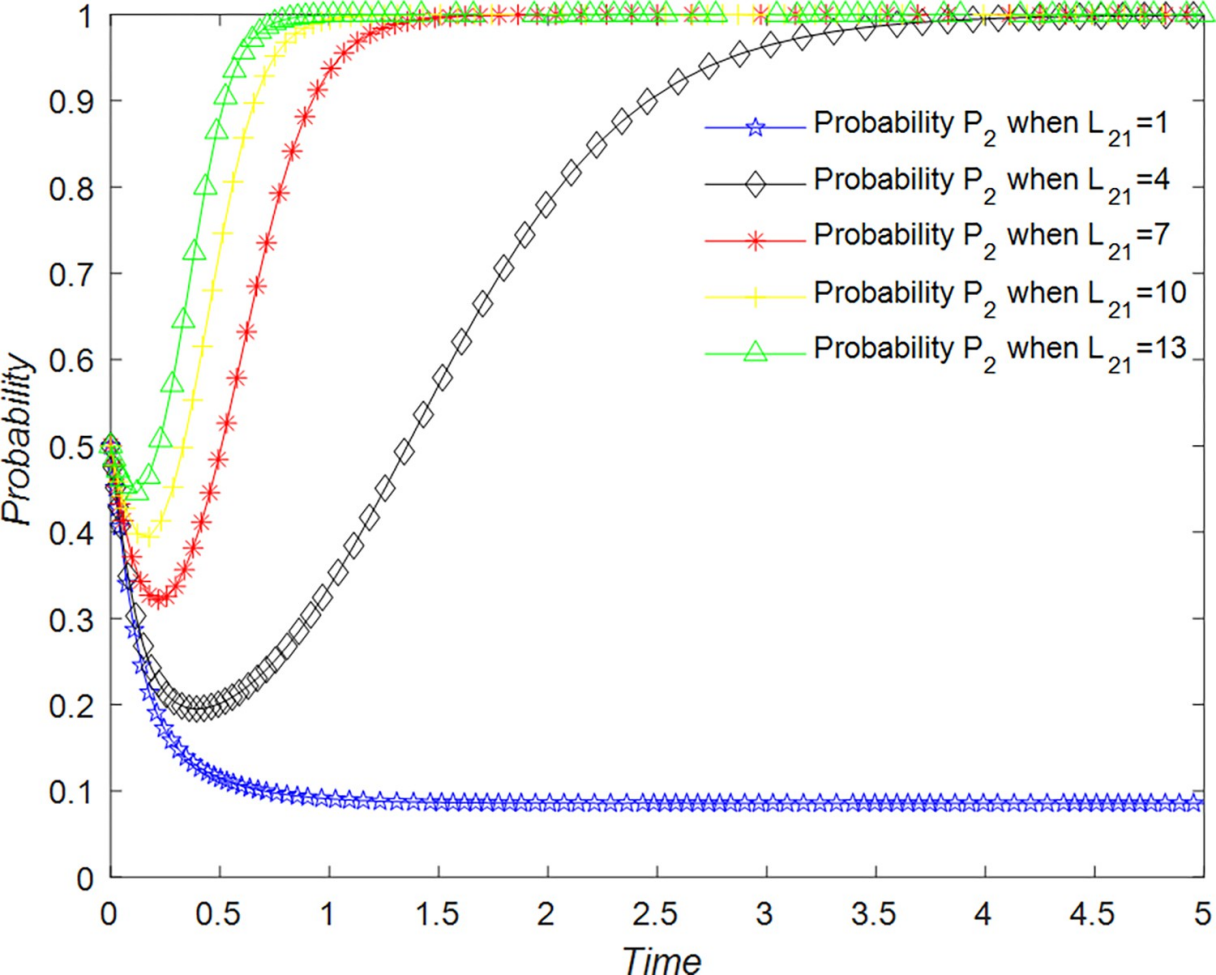
The influence of L_21_ on P_2_.

**Fig 38:**
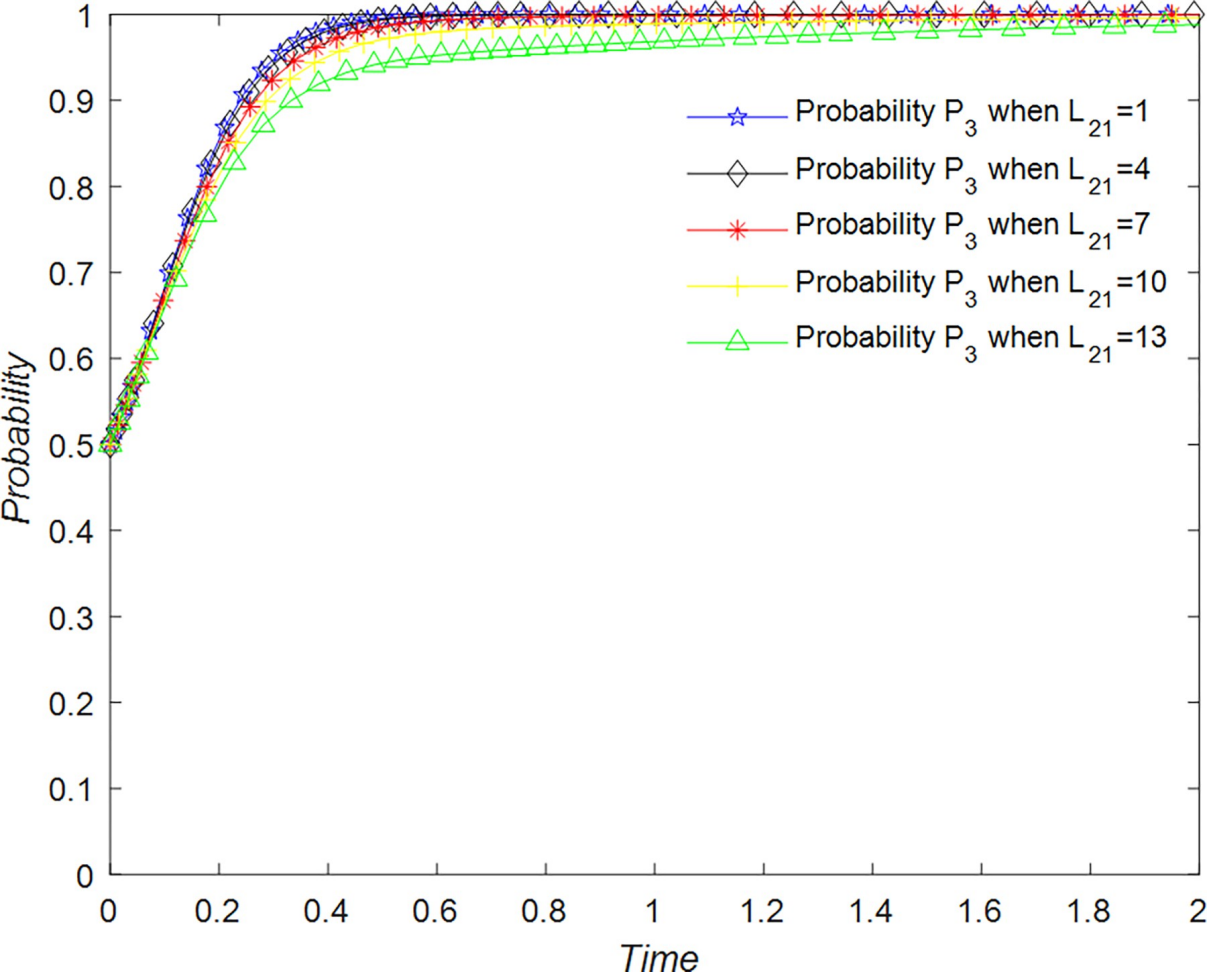
The influence of L_21_ on P_3_.

The government’s management of the B&B industry involves supporting the good B&Bs and exposing the bad practices of others, which can potentially lead to a loss of visitors for these B&Bs due to negative publicity. In this study, we consider different values of L21, specifically 1, 4, 7, 10, and 13, to analyze their impact on the simulation results. The effects of L_21_ on P_1_, P_2_, and P_3_ are shown in Figs 36–38, respectively.

Tourists have a wide variety of B&Bs to choose from in their destinations, and they have the opportunity to choose a well-run B&B to enhance their travel experience. Therefore, the individual loss of L_21_ from a single B&B has a minimal impact on tourists. As for the government, it acts as an overall manager to develop local rural revitalization businesses. The government’s focus is to ensure that the entire local market is thriving and can generate significant economic income. Therefore, L_21_ has little influence on the government’s decision regarding B&B management.

The impact of L_21_ is significant for B&B operators due to the competition between individual B&Bs in a given destination. B&Bs that cannot provide quality services are likely to lose customers to other B&Bs. Studies show that B&B operators initially choose to operate inferior facilities to avoid additional costs. However, as L_21_ increases, B&B operators are more likely to shift to a quality operations strategy. A larger L_21_ leads to a faster shift to a quality business strategy by B&B operators. When L_21_ is small, B&B operators may choose a lower cost business strategy. It can be concluded that a larger L_21_ plays a positive role in improving the management of the B&B industry.

#### Influence of loss of visitors (L_21_) caused by government regulation to B&Bs when they choose non-quality operations

**Fig 39:**
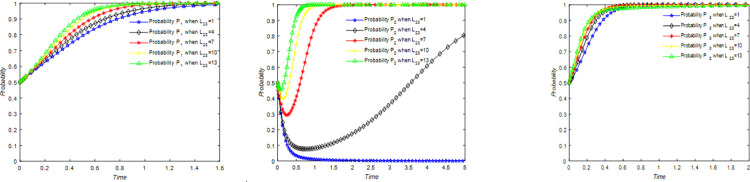
The influence of L_23_, on P_1_, on P_2_, on P_3_.

The government takes punitive measures, including fines, to encourage B&Bs to optimize their management when they adopt a non-quality business strategy. This may result in a loss for the B&Bs but a benefit for the government. In this study, the values of L_23_ were used as 1, 4, 7, 10, and 13 for the simulation analysis. Fig 39 illustrate the influence of L_23_ on P_1_, P_2_, and P_3_, respectively.

Tourists’ strategy choices are not influenced by government fines imposed on B&Bs. Instead, tourists tend to support the stable state of B&B industry optimization, and the larger the value of L_23_, the faster they move toward this stable state. This is because larger fines motivate B&Bs to solve their problems more efficiently, and B&B operators seek more advice from tourists, which leads tourists to support the optimization of the B&B industry management more quickly.

Initially, B&B operators may prioritize reducing operating costs over paying fines. Over time, however, there tends to be a gradual shift toward choosing a quality business strategy. This is because government fines increase the cost of choosing non-quality business strategies for B&Bs. Under the combined effect of the market and the government, B&B operators gradually come to believe that a quality business strategy is a better choice. However, if L_23_ is relatively small, it may take a long time for B&B operators to change their business strategies. An exceptionally small L_23_ may result in B&B operators continuing to choose non-quality business strategies.

The impact of L_23_ on the government would be small, and the government would quickly move into a steady state of managing the B&B industry. Although fines generate revenue for the government, the government does not prioritize this revenue. On the one hand, this revenue is a small percentage of the government’s total revenue. On the other hand, the fine is only a means for the government to regulate the behavior of local B&B operators and encourage them to improve the management of their B&Bs and provide quality services. The ultimate goal of the government is to promote rural revitalization and improve local economic development, not to generate revenue from fines imposed on B&B operators.

## Discussion, conclusion, managerial implication and practical implications

### Discussion, limitations and future work

#### Discussion

As an important component of rural tourism, rural B&Bs play a dual role, contributing to increased income in rural areas and facilitating the preservation and continuation of local culture. Analyzing the management and development of B&Bs allows the formulation of pragmatic and efficient strategies to optimize the management of rural B&Bs and promote the sustainable expansion of tourism. This paper aims to provide essential guidance and support for the future development of Yunnan’s rural revitalization efforts.

This paper proposes a comprehensive analytical framework for studying B&B reviews by incorporating advanced machine learning techniques, including the Bert model, CNN+BiLSTM, and CNN+BiGRU. The Bert model is used for topic analysis, while the CNN+BiLSTM and CNN+BiGRU models are used for sentiment analysis. This approach enables the identification of tourists’ key concerns through theme analysis and combines the results of sentiment analysis to provide a holistic understanding of the most pressing issues currently facing Yunnan B&Bs. The framework not only provides valuable insights into optimization strategies and corrective actions for the management of Yunnan B&Bs in the context of rural revitalization but also utilizes the latest objective website data. In particular, it uses a sentiment analysis model with a remarkable accuracy rate of 94%, surpassing the performance of most similar studies. This high level of accuracy ensures the accuracy, objectivity, and reliability of the research findings, making them highly valuable as reference points.

This study shows that the most important facets of tourists’ expectations of Yunnan B&Bs are the provision of passionate, high-quality services by B&B operators and the emotional experiences provided by folklore operators, underlining the importance of ’humane management’. On this basis, the primary way to optimize the management of Yunnan B&Bs is to vigorously improve service quality and attitude, while ensuring that basic requirements such as cleanliness and hygiene are met. Our speculation for these findings is as follows: The attraction of rural tourism lies in the natural landscapes and local traditions. Tourists want to enjoy the picturesque scenery, immerse themselves in local customs and traditions, and enjoy a leisure experience that is different from urban life. As a result, the physical facilities of B&Bs are not a major concern for tourists, who are primarily concerned with basic standards of cleanliness. However, B&B operators play a key role as purveyors of tourist information and serve as the primary conduit for tourists to participate in local customs. Therefore, tourists are particularly sensitive to the quality of service provided by B&B operators.

Furthermore, the analysis carried out in this framework underlines that the human element is the main influencing factor on issues within the rural B&B industry. Consequently, current management optimization efforts should prioritize the identification of key stakeholders involved in the process of improving the management of Yunnan’s B&Bs.

The stakeholders include tourists, B&B operators, and the government, who can choose from a range of strategies such as "support/not support", "quality/non-quality", and "management/non-management". All three stakeholders have limited rationality and cognitive constraints that affect their decision-making processes. Strategy choice is a dynamic process that is influenced by and affects the choices of the other two stakeholders. In addition, a dynamic evolutionary game model is introduced to analyze the replicator dynamics (RD) and evolutionary stable strategy (ESS) of the key stakeholders in the rural B&B industry. Finally, numerical simulations are conducted to explore the impact of various variables on the model. Through this model, this chapter provides insights into the evolutionary stable strategies of each stakeholder and the overall system with a focus on rural revitalization.

Based on our analysis, we have identified two main evolutionary stable strategies for the rural B&B industry. The first strategy, represented by the stability point (1, 0, 1), involves the government and tourists working to optimize B&B management, while B&B operators choose not to do so. The second strategy, represented by the stability point (1, 1, 1), involves all stakeholders working together to optimize B&B management. The dynamics of these strategies are influenced by the decisions of each stakeholder in the short run and the constraints of the evolutionarily stable strategies in the long run.

Tourists’ decisions are influenced by the emotional value offered by B&B operators (R_12_), incentives offered by the government (R_13_), and the potential loss of a good experience on their next trip (L_12_). B&B operators’ decisions are influenced by positive feedback from tourists about their quality operations (R_21_), government support and rewards (R_23_), the additional cost of implementing quality practices (C_21_), the potential loss of future customers (L_21_), and the risk of government fines for bad behavior (L_23_). Ultimately, B&B operators seek to maximize profits while minimizing costs. Government decisions are influenced by the potential loss of local revenue and economic development due to reduced tourism (L_3_).

The government’s decision to take a "management" strategy has a significant impact on whether B&B operators choose a "quality" strategy. It does not influence tourists’ choice of strategy. The government can encourage B&B operators to operate in good faith and build a positive reputation. It can also guide tourists to support rural tourism development by increasing rewards for those who do so. Once the government takes the "management" strategy, it promotes the process of B&B operators adopting a "quality" approach and increases the incentives for tourists to support rural tourism development. Ultimately, this leads to a balance of interests between the three parties involved.

According to the research, B&B operators often start with non-quality business strategies and adopt a "wait and see" approach, adjusting their strategies in response to market conditions and external factors. However, this behavior can have significant negative consequences as the market is highly competitive and even short periods of hesitation can result in significant financial losses.

#### Limitations

Optimizing the management of B&Bs in Yunnan is a complex and ongoing task that requires consideration of various factors by both the government and B&B operators. The subjective nature of tourist reviews makes it difficult to thoroughly analyze the content and direction of this optimization. This study focuses on data from Ctrip and does not include all online travel platforms, which may limit its applicability. In addition, the study does not take into account the differences between urban and rural areas and is limited to rural B&Bs in Yunnan Province.

#### Future work

The evolutionary game model proposed in this paper is an important step towards formalizing and understanding the complex interactions between different stakeholders in Yunnan’s B&B and rural tourism. However, it is still a theoretical starting point in the highly complex field of uncertainty research. Future research can extend the current model and refine the classification of residents based on specific characteristics and scenarios. Another potential direction is to integrate socio-demographic variables into the model’s utility function, including age, gender, income, education, and prior experience. In addition, B&B reviews are subjective and can be misleading, and further research can explore the use of next-generation digital technologies (e.g., blockchain, etc.) to improve the accuracy of review data and provide a solid foundation for investigating related issues. In addition, future research efforts should prioritize the exploration of green development strategies. It is imperative to emphasize the integral role of digital technology in green development as a focus for future research [69].

### Conclusion

The development of rural B&Bs in Yunnan has a significant impact on both rural economic growth and broader rural revitalization efforts. In this context, this paper uses the Bert model and applies machine learning techniques, including CNN+BiLSTM and CNN+BiGRU, to identify the most pressing issues facing Yunnan’s B&B sector. It then examines the stakeholders involved and introduces a three-party evolutionary game model to explore their interactions to optimize rural B&B management. The framework offers valuable optimization strategies and corrective pathways for Yunnan’s B&B management within the overarching framework of rural revitalization. The model explores the evolutionary stabilization strategies of each stakeholder and the system as a whole. It examines the stability of different equilibrium states and analyses the impact of exogenous variables on the decision-making processes of each stakeholder. This in-depth analysis provides key management insights that can help governments, local authorities and the public make informed decisions about rural accommodation management. In particular, to the best of our knowledge, this study represents the first attempt to conduct both perceptual and managerial analyses on the optimization of Yunnan’s B&Bs.

In terms of data, this study draws on recent information from online travel platforms, ensuring that the analyses are both current and compelling. In terms of addressing sentiment-related issues, this paper demonstrates innovation by using a machine learning model to explore the key challenges and stakeholders within the Yunnan B&B industry, while leveraging current data. In particular, the study employs the highly effective Bert model for topic extraction, and the accuracy of the sentiment analysis model employed surpasses that of traditional machine learning models. In terms of game analysis, this research extends the application of evolutionary games, marking the first use of such an approach in the context of Yunnan B&B management optimization. The main findings of this paper are outlined below.

Based on the topic analysis and sentiment analysis, it is evident that the main concern in contemporary rural folk lodges in Yunnan is to improve service attitude and quality while ensuring compliance with basic hygiene standards. The key stakeholders involved in optimizing the management of Yunnan’s B&Bs include the government, B&B operators, and tourists.

While theoretical considerations suggest the existence of eight possible equilibria, in practice, only two show the potential to be viable evolutionary game strategies, as revealed by our analysis. Each strategy embodies a different evolutionary result. The scenario (1, 0, 1) represents a situation in which both the government and tourists wish to witness the improved development of rural B&Bs. However, B&B operators are reluctant to improve their management standards, reflecting an immature stage in the development of Yunnan’s B&Bs. Conversely, the stabilizing strategy represented by (1, 1, 1) implies that by improving relevant policies and business models, it is possible to achieve a stable equilibrium in which all three parties actively support the development of rural B&Bs in Yunnan. In the long term, this alignment represents an ideal approach to promoting the development of rural tourism.

Several factors have a significant influence on the evolutionary trends, in particular the additional costs incurred by B&B operators in implementing quality business strategies and the economic consequences associated with the loss of potential tourists. In particular, the decision-making process of B&B operators is strongly influenced by fluctuations in these various factors. Given the key role of B&B operators in the optimization process, it is imperative to consider the benefits that accrue to them. It is also worth noting that, in the initial phase, B&B operators tend to opt for a low-quality business strategy. This is due to their general reluctance to invest in management optimization costs until they see tangible benefits.

### Managerial implication

The B&B industry plays a key role in generating local financial revenues and promoting rural revitalization and development. Therefore, local governments and relevant regulatory bodies need to actively support and monitor the operation and management of local B&Bs, thereby promoting standardization and improving the overall quality of the B&B sector. Firstly, B&B establishments should formulate operational and management strategies tailored to the current needs of tourists, ensuring the maintenance of a healthy and orderly business environment. In addition, relevant regulations, operational standards, and B&B management systems should be improved, building on the existing industry framework. At the same time, there is an urgent need to refine the inspection and assessment system for B&B operations and to implement appropriate penalties for non-compliance. In addition, local tourism departments should work with relevant regulatory organizations to establish a comprehensive regulatory framework for B&Bs, covering areas such as environmental protection, hygiene, food safety, and fire safety, all aimed at ensuring the overall operational quality of B&Bs. Second, local governments should provide financial and tax incentives to outstanding B&B operators as a tangible expression of encouragement and support.

Effective management is the linchpin for promoting the sustainable development of rural B&Bs. Therefore, local governments at all administrative levels must establish standardized management protocols. To this end, we recommend that local governments develop quality classification and evaluation standards for rural B&B services. These standards would serve to provide operators with explicit guidelines while empowering relevant authorities to strengthen their management, guidance, and evaluation functions. It is imperative to streamline multi-departmental management to mitigate cross-functional complications that could be detrimental to the healthy growth of rural B&Bs. To further raise the operational threshold of rural B&Bs, measures need to be taken to curb low-level redundancy in construction and operation. In addition, industry associations should be given more authority to play a more central role in guiding and harmonizing the quality development of rural B&Bs.

Yunnan’s rural B&Bs have a dispersed spatial layout but lack robust developmental linkages. To address this issue, it is necessary to focus on intensifying linkages and promoting the growth of an industry chain. Temporal and spatial chain linkages can be established by jointly bundling accommodation with scenic area tickets and by creating tailor-made tourism packages for different demographic segments, taking into account factors such as itineraries, culinary experiences, and length of stay. It is imperative to make use of the abundant rural resources by integrating picturesque landscapes, indigenous customs, folk traditions, and local specialties into the B&B chain, thus revitalizing rural areas.

### Practical implications

There is an urgent need to formulate funding policies and financial support mechanisms that encourage investment and innovation within the B&B industry. At the same time, it is essential to strengthen promotional and marketing efforts to raise awareness of the distinctive features and cultural characteristics of Yunnan’s B&B industry, both domestically and internationally. This can include the creation of a robust brand image and the implementation of comprehensive publicity campaigns across various media platforms, including social media, television, and print. It is also imperative to strengthen a robust assurance system for B&B development. This would involve enforcing compliance with industry standards and norms and improving the overall quality of B&B operations and services through the implementation of a regulatory framework.

To revitalize rural areas and facilitate effective communication with tourists, local governments should allocate resources to community outreach, education, and training initiatives for residents. These programs should be carefully tailored to the specific needs of the community, thereby minimizing the likelihood of conflict arising from tourism activities. It is also essential to improve the relationship between B&Bs and tourists, which can be achieved by optimizing customer feedback and evaluation channels. Regular training for B&B service staff, focusing on professionalism, etiquette, and courtesy, will further strengthen this relationship. The establishment of standardized protocols and benchmarks for B&B services serves as the basis for cultivating distinctive service styles and consistent offerings within each household. In maintaining the safety and well-being of guests, B&Bs must prioritize personal and property security, which includes comprehensive measures for epidemic control, food safety, fire safety, and traffic safety. In addition, addressing the psychological needs of tourists and offering specialized products can enhance the overall tourist experience.

In conclusion, the development of rural accommodation in Yunnan requires a comprehensive and sustainable approach that takes into account the cultural, environmental, and social dimensions of the region. This approach is essential to ensure that the B&B industry not only has a positive impact on the local economy and community but also preserves the region’s distinctive natural and cultural heritage.
